# Nuclear rupture induced by capillary constriction forces promotes differential effects on metastatic and normal breast cells

**DOI:** 10.1038/s41598-024-64733-x

**Published:** 2024-06-26

**Authors:** Julia Perea Paizal, Sam H. Au, Chris Bakal

**Affiliations:** 1 Department of Bioengineering, Imperial College London, London, SW7 2AZ UK; 2 Division of Cancer Biology, Chester Beatty Laboratories, Institute of Cancer Research, 237 Fulham Road, London, SW6 6JB UK; 3 Cancer Research UK Convergence Science Centre, Roderic Hill Building, Imperial College London, London, SW7 2BB UK

**Keywords:** Breast cancer, Stress signalling, Biomedical engineering

## Abstract

During metastatic dissemination, circulating tumour cells (CTCs) enter capillary beds, where they experience mechanical constriction forces. The transient and persistent effects of these forces on CTCs behaviour remain poorly understood. Here, we developed a high-throughput microfluidic platform mimicking human capillaries to investigate the impact of mechanical constriction forces on malignant and normal breast cell lines. We observed that capillary constrictions induced nuclear envelope rupture in both cancer and normal cells, leading to transient changes in nuclear and cytoplasmic area. Constriction forces transiently activated cGAS/STING and pathways involved in inflammation (NF-κB, STAT and IRF3), especially in the non-malignant cell line. Furthermore, the non-malignant cell line experienced transcriptional changes, particularly downregulation of epithelial markers, while the metastatic cell lines showed minimal alterations. These findings suggest that mechanical constriction forces within capillaries may promote differential effects in malignant and normal cell lines.

## Introduction

Metastasis accounts for 90% of cancer-related deaths worldwide^1^. During metastatic dissemination, cancer cells are shed from the primary tumour into the circulation as circulating tumour cells (CTCs), where they may encounter capillary beds they must traverse prior colonising a secondary organ. These capillaries have diameters often many-fold narrower than the diameter of CTCs. This makes capillary beds prime sites for the arrest of CTCs, which can remain entrapped until prior to initiating extravasation, which usually takes place 1–3 days after intravasation^2^. During this time, CTCs are exposed to mechanical forces, such as shear stress and compression, which can lead to intense cell deformation^3–5^.

Previous studies have shown that constricted migration experienced by cancer cells whilst in primary or secondary tumour sites or when extravasating, induces severe nuclear deformation, which can affect cell behaviour^6^. For instance, extreme nuclear deformation can lead to nuclear rupture, resulting in the mislocalisation of nuclear factors^7–10^, which can subsequently induce DNA damage^7,11–13^. Additionally, nuclear rupture leads inevitably to the exchange of nucleoplasmic content and cytosolic DNA, which can trigger the activation of the cGAS/STING pathway^11,13–15^. Nuclear deformation can also promote the formation of micronuclei^13^, which constitutes an additional source of cytosolic DNA^16^. Cytosolic DNA can promote an inflammatory state, epithelial-to-mesenchymal-transition (EMT)^17^, cell senescence^14,18,19^, stemness^20^ and drive metastasis^15,17^. It has also been proposed that extreme nuclear deformation can have an impact on cell cycle^10,21,22^ or induce changes in gene transcription^23–25^. Thus, there is evidence showing that nuclear deformation can drive more invasive phenotypes, inflammation, drug resistance and eventually lead to a worsened prognoses^17,25,26^.

Although some of these effects have been observed on cancer cells migrating through interstitial spaces, it remains unknowns whether they could also be triggered by mechanical forces in capillary beds since the number of studies focusing on the role of microcirculation on CTCs is very sparse as it is a quite challenging process to investigate. Yet, it has been reported that shear stresses within capillaries can induce morphological and molecular alterations^25,27^ and the activation of pro-survival mechanisms^28^. It is important to highlight that there are key differences between constricted migration through interstitial spaces and transit through capillaries. The latter is guided by fluid forces^4,27^ rather than chemoattractants or cell-directed migration^29^. Fluid forces, together with differences in pore and capillary dimensions and geometries, also affect the transit rates at which cells move within capillaries, which are shorter than those observed within interstitial spaces (seconds compared to hours or days)^10,13,30,31^. These differences in transit rate may also affect the cell viscoelastic properties^32^.

In this study, we sought to investigate whether the relatively short nuclear deformation that cells experience within capillaries could in the first place induce transient effects on cell behaviour, and whether these could ultimately get translated into persistent consequences that affect cancer progression. To address this question, we developed a high throughput microfluidic platform that replicates the geometry of small capillaries while enabling the transit of cells directed by fluid forces under physiological pressure rather than by a chemoattractant gradient. This platform enabled us to evaluate the effects of constrictions sizes on nuclear rupture, while enabling the collection of sufficient cells for downstream analysis. In this study we used a panel of triple negative breast cells with different phenotypes: the metastatic cell line MDA-MB-231 and its lung-metastatic derivative LM2^33^, which has a higher metastatic potential to the lungs, and the normal breast epithelial cell line MCF-10A. Their response after transiting constrictions was evaluated and it was observed that capillary transit induced transient cGAS/STING activation and promoted proliferation and the transient activation of inflammatory markers, such as NF-κB, STAT and IRF3. These changes were especially strong in the normal cell line MCF-10A. Moreover, MCF-10A experienced transcriptional changes and most of them resulted to be a downregulation of epithelial markers (such as cadherins, collagen, fibronectin, laminins, integrins or keratins, among others), rather than an upregulation of mesenchymal markers while the metastatic cell lines MDA-MB-231 and LM2 did not show downregulation of any these markers.

Overall, we observed that mechanical constriction forces induced by capillary constrictions led in the epithelial cell line MCF-10A to changes in gene expression that suggested the initiation of a transition from an epithelial to mesenchymal state and a more inflammatory phenotype (displayed by a transient upregulation of NF-κB, STAT and IRF3 and changes in pathways involved in humoral immune response, cytokine activity or regulation of an inflammatory response). In contrast, mesenchymal cancer lines were only transiently affected by the transit through the capillary device. We propose that transient capillary constrictions may engage EMT and inflammatory pathways in epithelial cells, but that mesenchymal cancer cells have already evolved to pass through capillaries with little effects on fate determination.

## Results

### Engineering of a high-throughput capillary-on-a-chip microfluidic platform

**Figure 1:**
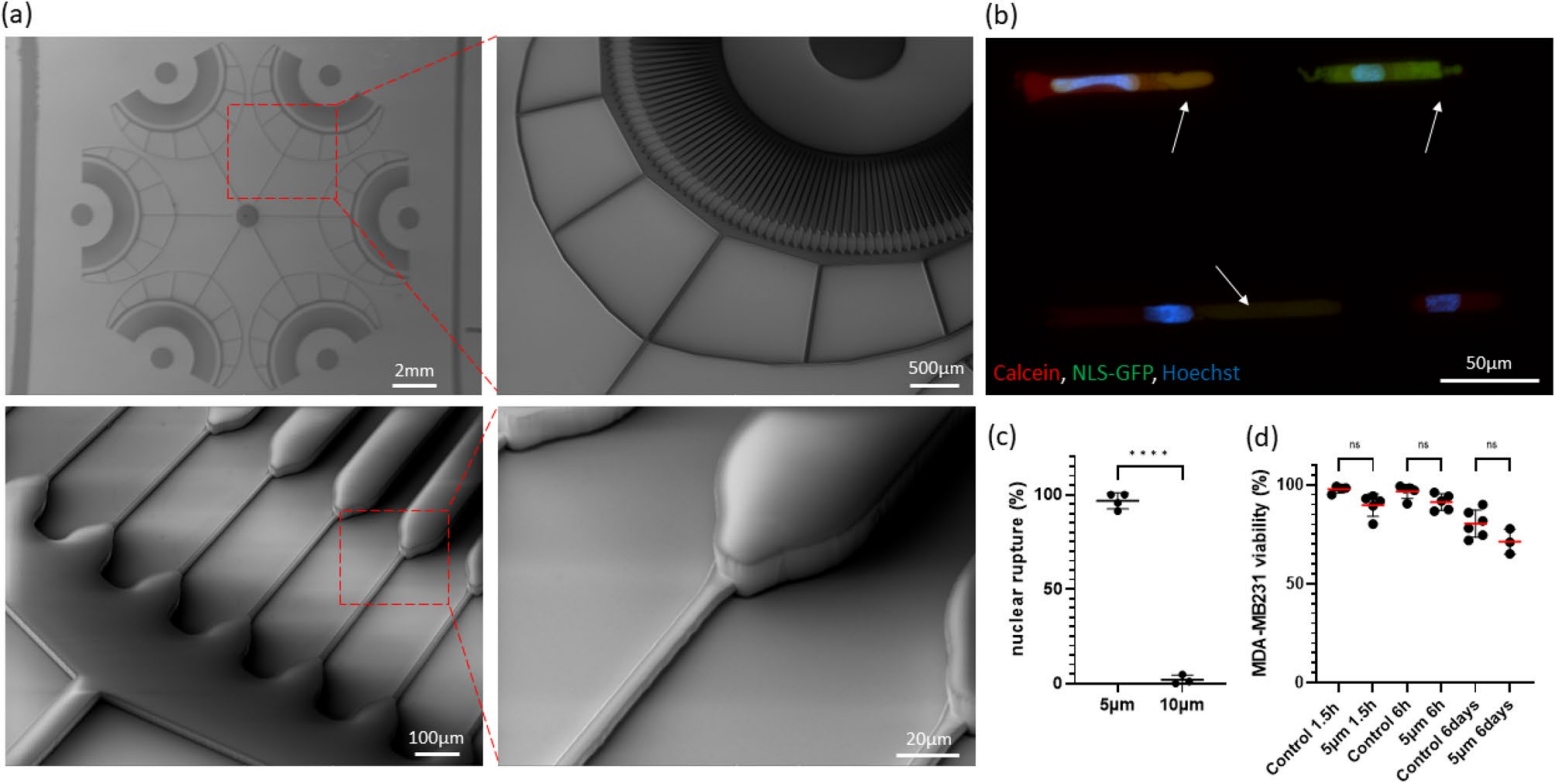
Effects of capillary constriction forces on nuclear envelope rupture and viability. (**a**) Scanning electron micrographs of a “snowflake” capillary-on-chip platform comprised of 5 × 5 µm^2^ constrictions of 150 µm length. Each chip comprises 510 constrictions, distributed throughout 6 inlets (85 constrictions per inlet). (**b**) Fluorescence image of NLS-GFP (green) MDA-MB-231 cell experiencing nuclear rupture while transiting a 5 × 5 µm^2^ capillary constriction under physiological pressures of 7–33 cm H_2_O. The cytoplasm was detected with Calcein Red-AM (red) and the nucleus with Hoechst (blue). White arrows indicate cells experiencing NE rupture. (**c**) Fraction NLS-GFP MDA-MB-231 cells experiencing nuclear envelope rupture while transiting 5 × 5 µm^2^ and 10 × 10 µm^2^-constrictions under physiological pressure (7–33 cmH_2_O). Each dot represents an individual replicate (percentage of cells that experienced nuclear rupture when transiting the same microfluidic device). A total of n = 77 cells for the 5 µm constrictions and n = 195 for the 10 µm constrictions were quantified. (**d**) Cell viability (%) of MDA-MB-231 1.5 h, 6 h and 6 days after transiting 5 × 5 μm^2^ constrictions compared to cells that did not transit the constriction (controls). Each dot represents a biological replicate, in which a minimum of 500 cells were quantified. Comparisons among groups were performed by unpaired *t* test, two-tailed (t = 46.48, DF = 6, *p < 0.05, **p < 0.01, ***p < 0.001, ****p < 0.0001).

To mimic the effects of capillary constriction forces, microfluidic “snowflake” platforms that feature capillary constrictions were engineered using standard photolithography techniques in polydimethylsiloxane bonded to borosilicate glass. Each microfluidic platform had 6 inlets each of these were upstream to 85 microchannels constrictions of 5 × 5 × 150 µm^3^ or 10 × 10 × 150 µm^3^ (width × height × length). The narrower constriction diameters fell within the physiological range observed in human capillaries, which typically range from 5.91 ± 1.30 µm in diameter^34^. Microfluidic devices were fabricated with two layers: the first one comprising the constrictions and the second one containing the inlets and collector channels (Fig. 1a). Microfluidic platforms were operated under physiological hydrostatic pressures (7–33 cmH_2_O)^35^, and cell velocities fell within the range of 12.57 ± 15 µm/s, consistent with previous studies replicating physiological capillary velocities^36^ (Fig. S1a)

### Effects of capillary constriction forces on nuclear envelope rupture

To evaluate whether cancer cells experience nuclear envelope rupture (NE) while transiting capillaries, MDA-MB-231 and MCF-10A cells tagged with green fluorescent protein conjugated to a nuclear localisation signal (NLS-GFP) were introduced in the capillary-on-a-chip platforms and NE rupture events were quantified on NLS-GFP MDA-MB-231 and NLS-GFP MCF-10A cells transiting the channels. NE rupture events were identified by GFP signal leakage into the cytoplasm, which was co-localised by staining the cell with Calcein Red-AM (Fig. 1b, Fig. S2c). On average, 96.60% ± 4% (n = 77 cells) MDA-MB-231 cells that transited 5 × 5 µm^2^ constrictions experienced nuclear rupture, whereas only 1.95% ± 2% (n = 195 cells) of the MDA-MB-231 that transited 10 × 10 µm^2^ showed NE rupture (Fig. 1c). NLS-GFP MCF-10A transiting 5 × 5 µm^2^ constrictions also experienced nuclear rupture (94.70% ± 7%, n = 149) (Fig. 1b). NE rupture events were observed when cell nuclei first encountered the constriction and within the constriction (Fig. S1b,c). Nuclear compartmentalisation was not recovered immediately upon existing the constriction, as 66.59% ± 3% of MDA-MB-231 (n = 129 cells) and 85.14% ± 9% of MCF-10A (n = 71) presented cytoplasmic NLS-GFP signal 30 min after transiting constrictions (Fig. S1d,e). Our results suggest that in our model, only 5 × 5 µm^2^ constrictions induced nuclear rupture on cells transiting capillaries, what led us to focus on 5 × 5 µm^2^ constrictions in the following experiments. These results are consistent with previous studies showing that nuclear size contributed to hydrodynamic resistance only at 5 µm constrictions or smaller^36^ and that NE rupture increases with a reduction of the pore size^13^.

### Effects of mechanical constriction forces in cell viability

Next, we investigated whether mechanical constriction forces affected cell viability. MDA-MB-231 remained viable for 1.5 h (89.7% ± 5%), 6 h (91.27% ± 4%) and 6 days (71.29% ± 6%) after transiting through 5 × 5 µm^2^ constrictions as judged by Calcein-AM/PI staining (Fig. 1d). MCF-10A cells also remained viable 6 days after transiting 5 × 5 µm^2^ constrictions (Fig. S2a). Thus, while the majority of cells passing through the capillaries experienced nuclear rupture, this had little effect on long-term viability.

### Effects of mechanical constriction forces on proliferation

**Figure 2:**
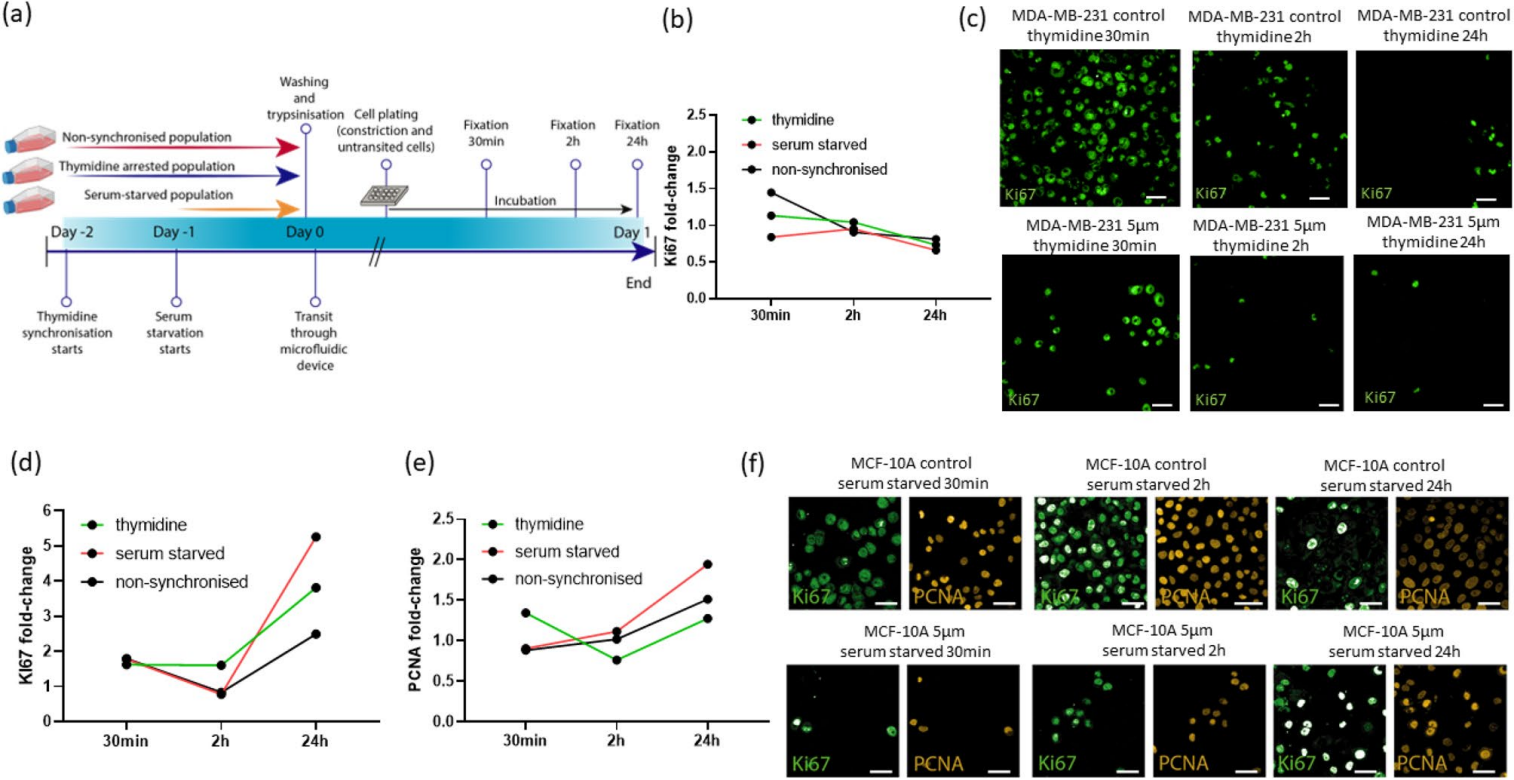
Effects of mechanical constriction forces on proliferation on MDA-MB-231 and MCF-10A cells. (**a**) Schematic illustration of the experimental timeline. (**b**) Ki67 fold-change of nuclear average intensity in MDA-MB-231 cells 30 min, 2 h and 24 h after transiting the constriction with respect to cells that did not transit the constriction. Cells were synchronised in two different phases of the cell cycle: G1/early S (thymidine synchronised) and G0 (serum starved for 24 h) and compared to non-synchronised cells. (**c**) Immunofluorescence confocal images of Ki67 (green) of MDA-MB-231 cells synchronised with thymidine and fixed 30 min, 2 h and 24 h after transiting the constriction, compared to untransited controls. Scale bar: 40 µm. (**d**) Ki67 and (**e**) PCNA fold-change of nuclear average intensity in MCF-10A cells 30 min, 2 h and 24 h after transiting the constriction with respect to cells that did not transit the constriction. Cells where synchronised in two different phases of the cell cycle: G1/early S (thymidine synchronised) and G0 (serum starved for 24 h) and compared to non-synchronised cells. (**f**) Immunofluorescence confocal images of Ki67 (green) and PCNA (yellow) of MCF-10A cells serum-starved and fixed 30 min, 2 h and 24 h after transiting the constriction, compared to control. Scale bar: 40 µm. Within each cell line, comparisons among groups were performed by one-way ANOVA (Tukey multiple comparison test, *p < 0.05, **p < 0.01, ***p < 0.001, ****p < 0.0001).

We next investigated whether constriction forces affected cell proliferation of cancer and normal breast cell lines, MDA-MB-231 and MCF-10A. Since replicating cells have larger nuclei as a result of the duplicated genetic material and a different stiffness because of the decondensed chromatin^37^, we synchronised the cells to early stages of the cell cycle to ensure we had a more homogeneous cell population with nonreplicated genomes (2N) prior to passing them through the constrictions. Cells were synchronised using two different methods: double thymidine block arrest at G1/S; and serum starvation at G0 (Fig. S3). Upon transit, cells were collected and fixed 30 min, 2 h and 24 h after transiting the constriction (Fig. 2a) and proliferation was assessed by quantifying Ki67 nuclear signal in cells that had transited the constriction and controls (Fig. 2b,d and Fig. S4). Additionally, the MCF-10A cells that were used in this experiment were tagged and quantified for PCNA, a protein whose expression peaks in G1/S^38^ (Fig. 2e and Fig. S4). Analysing the fold-change of nuclear Ki67 after capillary transit when compared to controls, we observed an increase in the thymidine synchronised population and in the asynchronous population 30 min after transiting the constriction. However, 24 h post-transit the nuclear intensity signal was lower than control groups (fold-increase lower than 1) in the three populations (thymidine synchronised cells, serum-starved cells and asynchronous cells). On the contrary, in MCF-10A cells the Ki67 and PCNA nuclear intensity signal remained higher than controls 24 h after transiting the constriction in the three populations (Fig. 2d,e). These results suggest that constriction forces promoted a transient increase of proliferation in both cancer and healthy cell lines, and that this effect was sustained in MCF-10A cells. These observations imply that mechanical constriction forces alone could promote proliferation in MCF-10A cells.

### Effects of mechanical constriction forces in cell morphology

**Figure 3:**
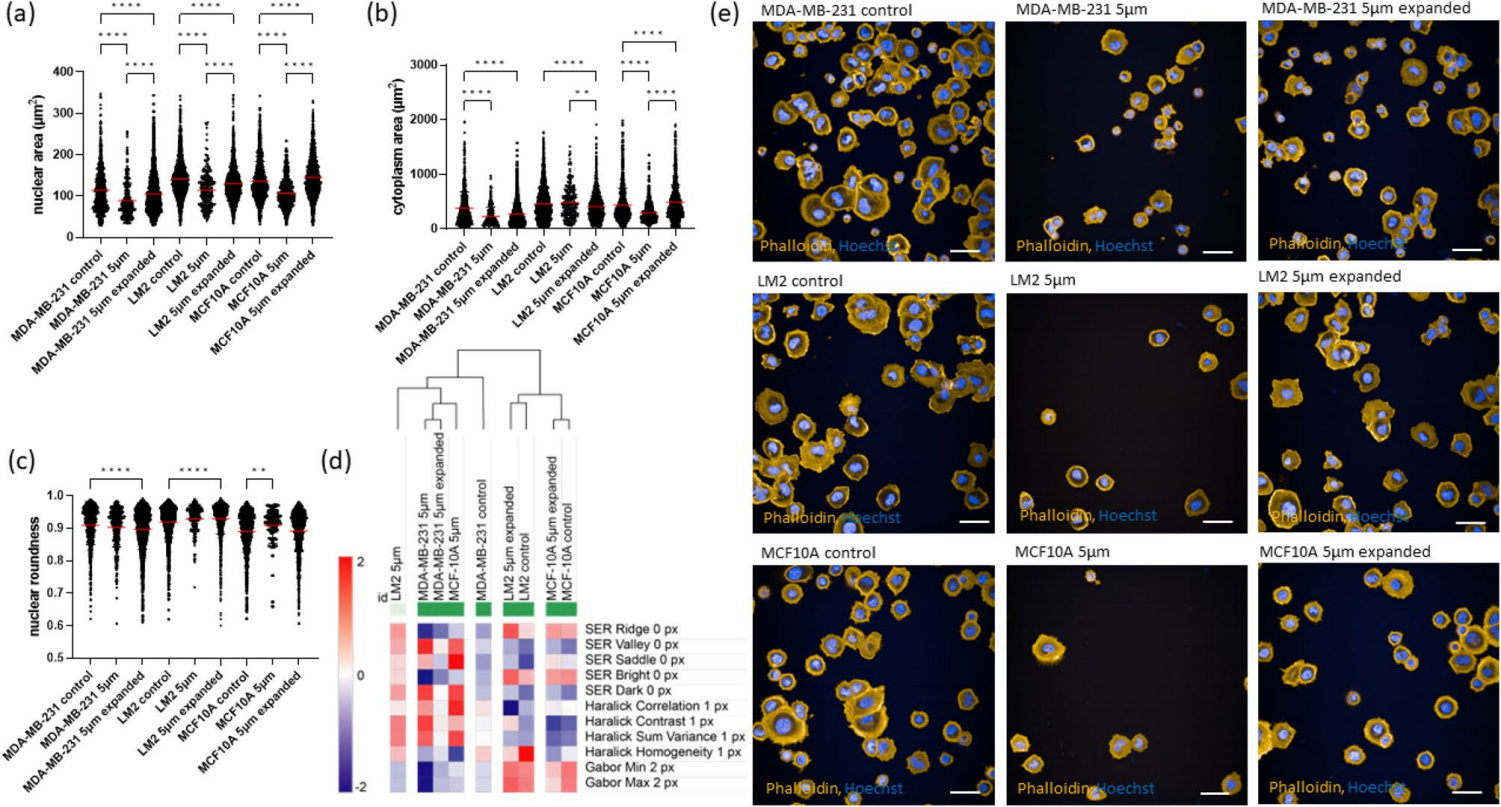
Effects of mechanical constriction forces on cell morphology. (**a**) Nuclear area (μm^2^) by segmenting the cells using Hoechst, (**b**) cytoplasm area (μm^2^) from segmenting the cell with Phalloidin, (**c**) nuclear roundness. The group “5 µm” refers to cells that have transited through 5 × 5 µm^2^ constrictions, the group “5 µm expanded” refers to cells that have transited 5 × 5 µm^2^ constrictions and have been expanded for 2–5 passages to provide them sufficient time to recover and the group “control” refers to cells that have not transited the constriction. Each dot represents a cell. For each group, means are highlighted in red. (**d**) Heatmap of tubulin textures (SER, Haralick and Gabor) across the cell body and hierarchical clustering of the panel of cells after transiting 5 µm constrictions, expanded cells and controls. (**e**) Immunofluorescence images from confocal microscopy of actin (Phalloidin) and nuclei (Hoechst) of MDA-MB-231, LM2 and MCF-10A cells. Scale bar: 40 µm. Within each cell line, comparisons among groups were performed by one-way ANOVA (Tukey multiple comparison test; *p < 0.05, **p < 0.01, ***p < 0.001, ****p < 0.0001).

Since we observed that transit through constrictions resulted in nuclear envelope rupture (Fig. 1 and Fig. S2), we then investigated if the leak of material from the nucleus into the cytoplasm and the extreme deformation observed in the cell body while cells transited constrictions would affect nuclear and cytoplasmic morphology. To determine if constrictions affected cell morphology in 2D, the metastatic breast cell line MDA-MB-231, its lung metastatic variant MDA-MB-231 LM2 and the healthy breast cell line MCF-10A were collected after transiting 5 × 5 µm^2^ constrictions and expanded for 2–5 passages to provide them sufficient time to recover and evaluate whether the effects of constriction forces were persistent. These cells were compared to cells that just transited the constriction 30 min before and to cells that did not transit the constriction (control). Cells were stained and imaged by high-throughput microscopy. Upon transit, both nuclear and cytoplasmic area decreased (P < 0.0001) in the three cell lines (Fig. 3a,b,e), except for the cytoplasm in LM2. In the expanded cell lines MDA-MB-231 and LM2, both the nucleus and the cytoplasm had a reduction in area compared to their respective controls (8% nuclear area decrease for both MDA-MB-231 and LM2, 27% cytoplasm area decrease for MDA-MB-231 and 10% cytoplasm area decrease for LM2), whereas the MCF-10A cell line completely recovered and reached the original nuclear and cytoplasmic area. Nuclear roundness was not affected (Fig. 3c). We then decided to monitor closer the cytoplasm and nuclear area loss in MDA-MB-231 and MCF-10A by tracking their morphology 2 h and 24 h after cells transited the constriction. 24 h post-transit both the nucleus and cytoplasm area remained smaller than the control in MDA-MB-231 and MCF-10A cells (22% nuclear area loss and 26% cytoplasm area loss in MDA-MB-231 and 35% nuclear area loss and 9% cytoplasm area loss in MCF-10A) (Figs. S5a,b and S6a,b). MCF-10A cells had a decrease in nuclear/cytoplasm area ratio 24 h post-transit (Fig. S6c), suggesting that the area loss in the cytoplasm is larger than the nuclear area loss. On the contrary, MDA-MB-231 cells had similar nuclear/cytoplasmic ratios (Fig. S6c). Plotting the correlation between nuclear and cytoplasm area suggested that constrictions reduced both nucleus and cytoplasm area in both normal and cancer cells but also that possibly some selection mechanism might take place within constrictions since cells with very large nucleus and cytoplasm were not found post-transit (Figs. S5, S6d–f). Overall, these results indicate that constriction forces transiently affected cell morphology in normal and metastatic cell lines, and that the loss of nuclear and cytoplasmic area was persistent in MDA-MB-231 and LM2.

We then examined whether constrictions affected cytoskeleton microtubules organisation by evaluating changes in tubulin texture features (SER, Haralick and Gabor) across the cell body. A lower SER ridge value, suggestive of a decrease in tubulin filaments alignment, was observed in MDA-MB-231 and MCF-10A cells after transiting constrictions (Fig. 3d). Moreover, hierarchical clustering of the tubulin texture features clustered in different groups cell lines that had transited constrictions and untransited controls, suggesting changes in tubulin organisation upon capillary transit. In the MDA-MB-231 cell line, the texture features in the expanded counterparts were more similar to those of MDA-MB-231 cells that had transited constrictions, suggesting persistent changes in tubulin organisation in this cell line (Fig. 3d).

To determine if constrictions affected cell aggregation/morphology in 3D, we used cells that passed through the constriction to form spheroids, which were embedded in collagen and cultured for 7 days. No differences in spheroid area or protrusions were observed between spheroids made of cells that had transited the constriction and control (Figs. S7, S8), suggesting that constriction forces did not affect 3D morphogenesis.

### Effects of mechanical constriction forces in nuclear lamins

**Figure 4:**
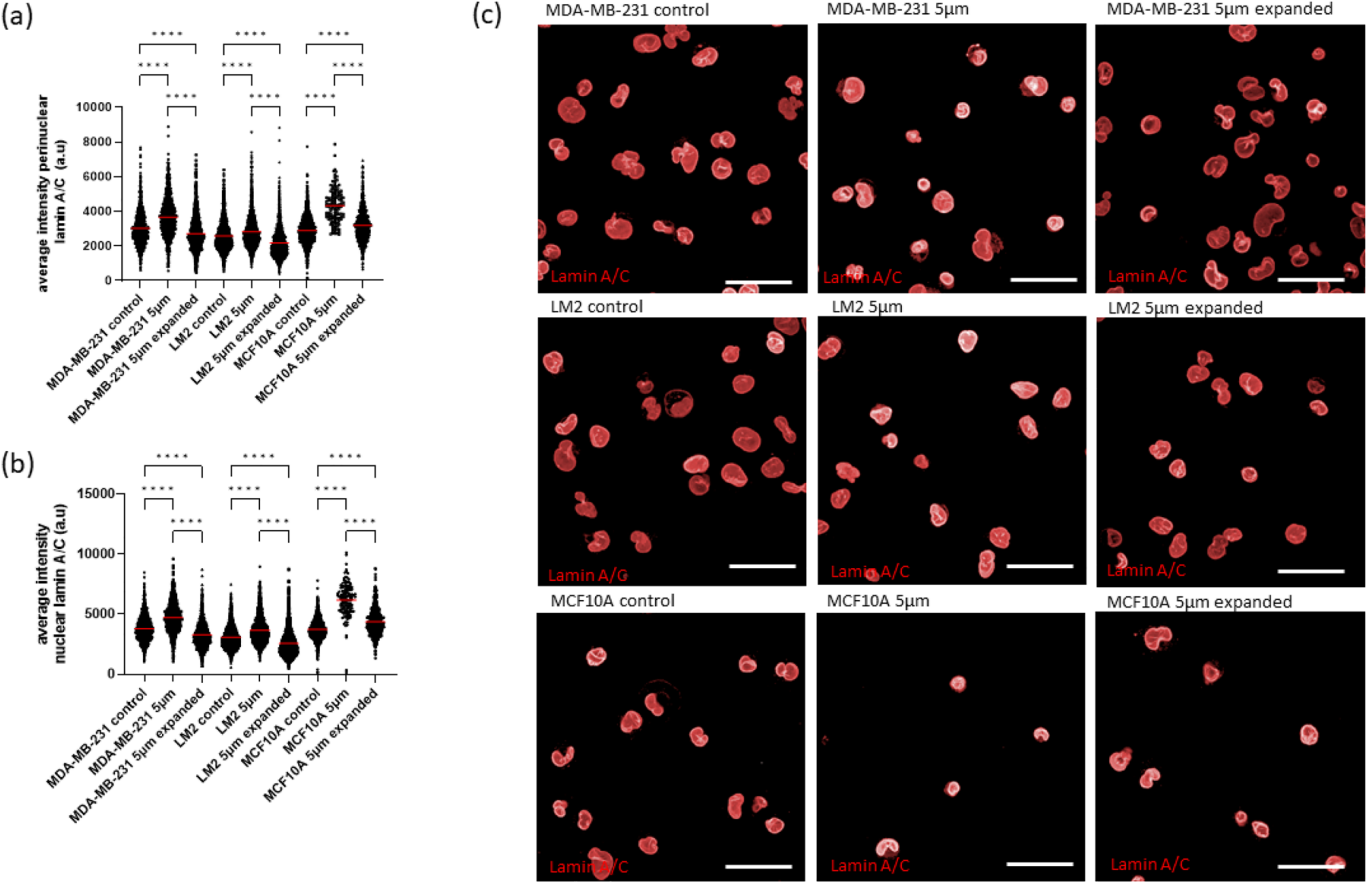
Effects of capillary constriction forces on the expression of nuclear lamins. (**a**) Lamin A/C perinuclear and (**b**) nuclear average fluorescent intensity from antibody staining. Each dot represents a cell. For each group, means are highlighted in red. (**c**) Immunofluorescence images from confocal microscopy of lamin A/C in MDA-MB-231, LM2 and MCF-10A cells. The group “5 µm” refers to cells that have transited through 5 × 5 µm^2^ constrictions, the group “5 µm expanded” refers to cells that have transited 5 × 5 µm^2^ constrictions and have been expanded for 2–5 passages to provide them sufficient time to recover and the group “control” refers to cells that have not transited the constriction. Scale bar: 40 µm. Within each cell line, comparisons among groups were performed by one-way ANOVA (Tukey multiple comparison test; *p < 0.05, **p < 0.01, ***p < 0.001, ****p < 0.0001).

As constriction forces induce nuclear envelop rupture (Fig. 1 and Fig. S2), we then sought to investigate nuclear membrane integrity on these same panel of cells after they transit 5 µm constrictions by measuring perinuclear intensity of lamin A/C, whose levels have been previously correlated with nuclear stiffness and nuclear viscoelasticity^39,40^. Lamin A/C perinuclear and nuclear intensity levels increased in all cells (MDA-MB-231, LM2 and MCF-10A) after transit through constrictions (P < 0.0001). This increase was more pronounced in MCF-10A (1.49-fold increase) and in MDA-MB-231 (1.21-fold increase) than in LM2 (1.09-fold increase). However, lamin A/C intensity returned to baseline after cells were given time to recover, demonstrating the effect of constrictions on lamin A/C was transient (Fig. 4).

We observed that constrictions altered the organisation of lamin A/C (“folding and wrinkles”) (Fig. S9a). A linear classifier using only lamin A/C features (intensity and texture) was trained with MDA-MB-231 and MCF-10A cells that had transited the constriction and control cells (Fig. S9b). The linear classifier trained with MCF-10A cells performed better than the classifier trained with MDA-MB-231, reflected by a higher metrics in the quality of the separation (referred in Fig. S9 as “goodness”). The quality of the separation was based on the signal to noise ratio of the distance of the training points from the classifier line. The linear classifier trained with MCF-10A cells was able to classify correctly 95.18% of the cells that had transited the constriction and 91.16% of the control cells. Moreover, 81.39% of the MCF-10A expanded cells were classified into the control group (Fig. S9c). The top properties required by the linear classifier to separate MDA-MB-231 that had transited the constriction from the control were texture features (SER ridge and SER bright), whereas for the MCF-10A cells the top features were mean intensity levels (cytoplasm, nucleus and perinuclear region mean intensity). Plotting cytoplasm intensity levels and nuclear SER ridge textures (Fig. S9d) confirmed that intensity levels could largely distinguish MCF-10A cells that had transited the constriction from control cells, whereas a set of textures features was needed to separate MDA-MB-231 cells.

To further investigate the role of lamins in NE rupture, NLS-GFP MDA-MB-231 cells were silenced for LMNA or LMNB1 in MDA-MB-231 cells (Fig. S10a,b) and compared to wild type cells. The frequency of NE rupture in these cells while transiting 5 × 5 µm^2^ constrictions was evaluated (Fig. S10c), revealing a slight increase in NE rupture in siLMNA cells (98.1% when compared to 96.6% of wt cells). siLMNA cells also had a stronger activation of the cGAS/STING pathway than wt cells (1.60-fold increase in the intensity of cytoplasmic dsDNA, 1.57-fold increase in cytoplasmic cGAS and 1.40-fold increase in perinuclear STING) (Fig. S10d). However, fold-increase of the nuclear fluorescent intensity signal of the canonical NF-κB p65 protein and the non-canonical NF-κB p100 protein was not higher than in wild type cells, suggesting that siLMNA cells do not have a stronger activation of the NF-κB pathway. Interestingly, siLMNB1 cells had similar levels of activation of the cGAS/STING pathway to wild type cells, suggesting that in our model, lamin A/C and not lamin B1 promoted a stronger activation of the cGAS/STING pathway in MDA-MB-231 cells, which will be further explored in the next section.

Overall, these results suggest that mechanical constriction forces induced a transient stiffening of the nucleus and a strengthening of the nuclear envelope by increasing LMNA expression, especially in the cell line MCF-10A. Moreover, constrictions changed mostly the organisation of lamin A/C in MDA-MB-231 (quantified by changes in texture), whereas in MCF-10A lamin A/C levels were more affected.

### Effects of mechanical constriction forces on the activation of the cGAS/STING pathway

**Figure 5:**
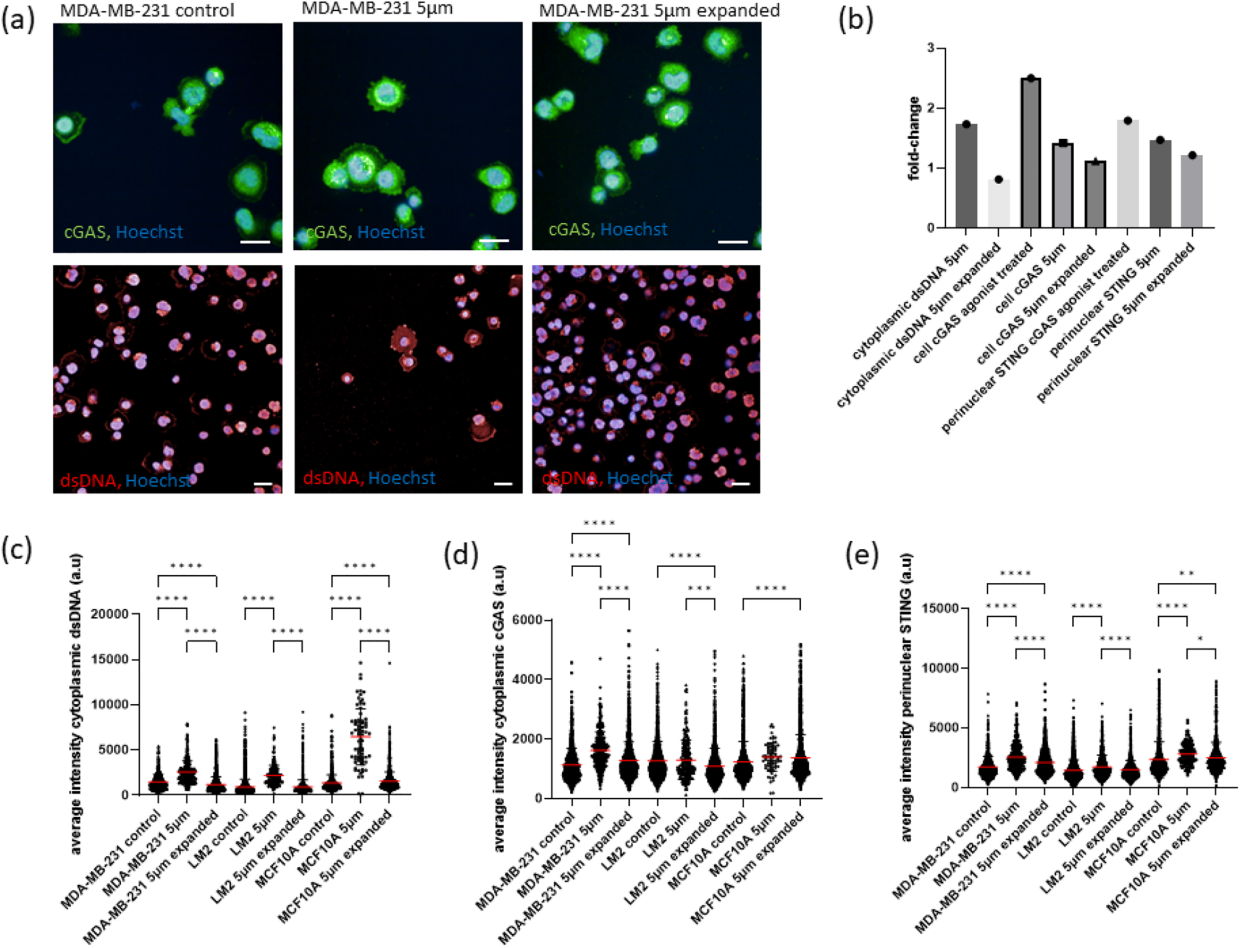
Effects of capillary constriction forces on the activation of the cGAS/STING signalling pathway. (**a**) Immunofluorescence confocal images of cGAS (green) and dsDNA (red) in MDA-MB-231 cells. The group “5 µm” refers to cells that have transited through 5 × 5 µm^2^ constrictions, the group “5 µm expanded” refers to cells that have transited 5 × 5 µm^2^ constrictions and have been expanded for 2–5 passages to provide them sufficient time to recover and the group “control” refers to cells that have not transited the constriction. Scale bar: 25 µm, (**b**) fold-change in the average intensity of cytoplasmic cGAS, perinuclear STING, and cytoplasmic dsDNA of MDA-MB-231 cells. Fold-change of cells treated with the cGAS agonist G3-YSD was calculated by comparing their intensity levels to the intensity levels of untreated cells. Fold-change of each group was calculated using the average of at least n = 200 cells. (**c**) dsDNA cytoplasmic average intensity, (**d**) cGAS cytoplasmic average intensity and (**e**) STING perinuclear average intensity from antibody staining in the cell lines MDA-MB-231, LM2 and MCF-10A. Each dot represents a cell. For each group, means are highlighted in red. Within each cell line, comparisons among groups were performed by one-way ANOVA (Tukey multiple comparison test; *p < 0.05, **p < 0.01, ***p < 0.001, ****p < 0.0001).

As nuclear envelope rupture has been previously reported to trigger the cGAS/STING pathway when cells undergo constricted migration^11,13–15^, we sought to investigate whether cells transiting 5 µm constrictions directed by fluid flow caused activation of this pathway. The activation of the cGAS/STING pathway was assessed by measuring in our panel of triple negative breast cells the signal intensity of cytoplasmic dsDNA, cytoplasmic cGAS and perinuclear STING (Fig. 5)^17^. To assess the sensitivity and specificity of cGAS and STING antibodies, MDA-MB-231 cells were first treated with the cGAS agonist G3-YSD, what resulted in a 2.40-fold increase in cGAS cytoplasmic intensity and a 1.79-fold increase in STING perinuclear intensity (Fig. 5b). A 1.42 and 1.47-fold increase intensity signal in cGAS and STING was observed on MDA-MB-231 cells transiting 5 µm constrictions (Fig. 5b).

In the panel of breast cancer cells, a significant increase in cytosolic dsDNA, cGAS and STING was observed across the cell lines, but they returned to baseline levels after expanding them (Fig. 5c–e). Notably, the intensity of dsDNA increased by 4.7-fold after transit, compared to the 1.73-fold increase of MDA-MB-231 and 2.31-fold increase of LM2 cells, suggesting that MCF-10A are more likely to experience nuclear rupture while transiting constrictions. These results suggest that constriction forces induced transient cGAS activation in the three cell lines as values tend to return to baseline levels in the expanded cell lines.

### Effects of mechanical constriction forces in the activation of an inflammatory response

**Figure 6:**
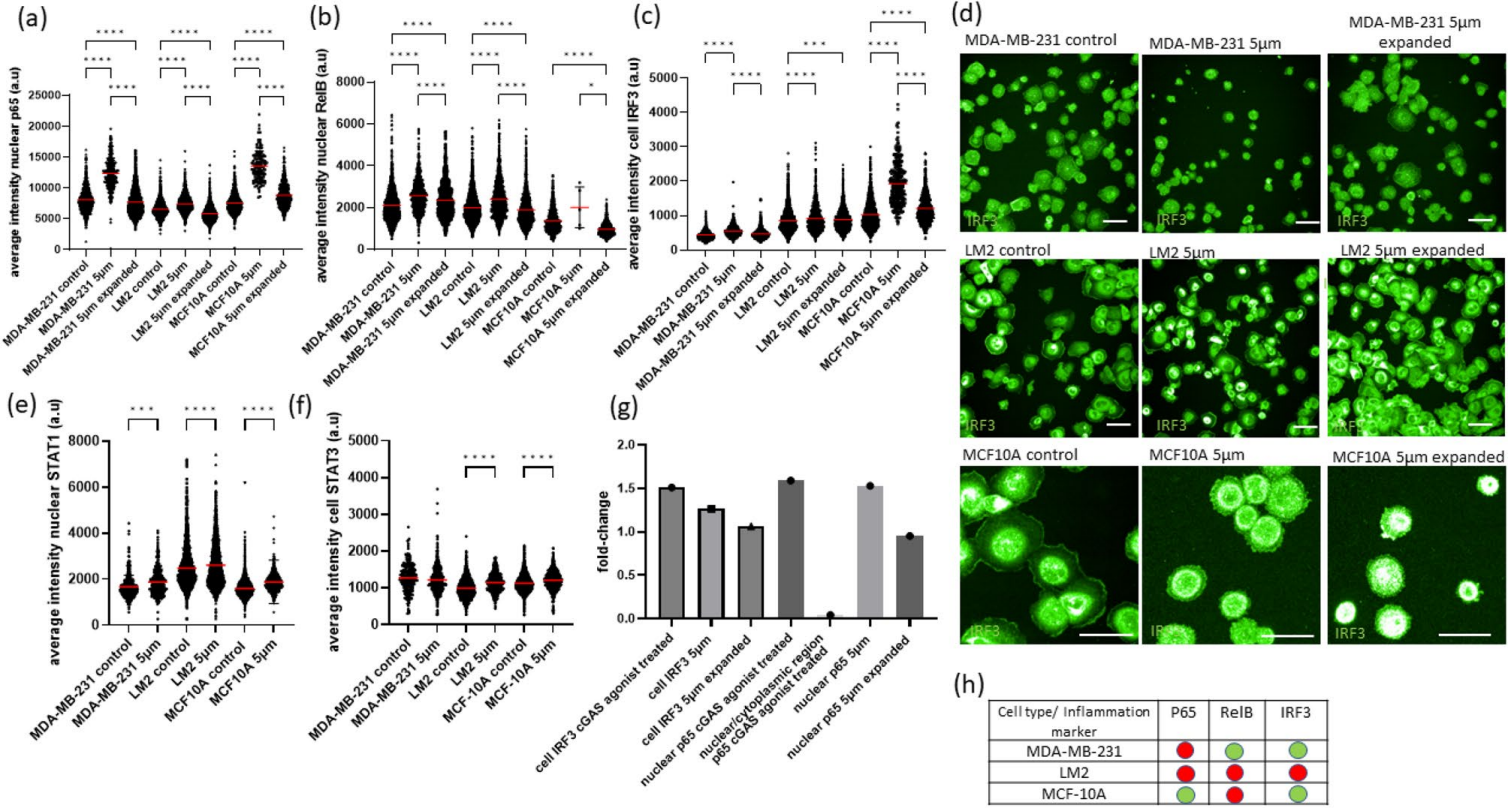
Effects of capillary constriction forces on the activation of inflammatory factors. (**a**) Results from antibody staining of nuclear average intensity of p65 (canonical NF-κB) and (**b**) nuclear average intensity of RelB (non-canonical NF-κB). (**c**) IRF3 cell average intensity, (**d**) immunofluorescence images from confocal microscopy of IRF3 after antibody staining. Scale bar: 40 µm, (**e**) nuclear average intensity of STAT1 and (**f**) cell average intensity of STAT3 of MDA-MB-231, LM2 and MCF-10A cells. The group “5 µm” refers to cells that have transited through 5 × 5 µm^2^ constrictions, the group “5 µm expanded” refers to cells that have transited 5 × 5 µm^2^ constrictions and have been expanded for 2–5 passages to provide them sufficient time to recover and the group “control” refers to cells that have not transited the constriction. Each dot represents a cell. For each group, means are highlighted in red. (**g**) Fold-change in the average intensity of cellular IRF3 and nuclear p65. Fold-change of cells treated with the cGAS agonist G3-YSD was calculated by comparing intensity levels of cells after treatment to intensity levels of untreated cells. For each group, a minimum of n = 200 cells were quantified. (**h**) Summary of inflammatory markers that presented a sustained response, indicated by signal increases that remained above 5% over time in the expanded cell lines (in green). Within each cell line, comparisons among groups were performed by one-way ANOVA (Tukey multiple comparison test; *p < 0.05, **p < 0.01, ***p < 0.001, ****p < 0.0001).

Since cGAS/STING has been previously reported to trigger an inflammatory response^17,18,41,42^, we assessed whether our panel of cells would transition into an inflammatory state induced by mechanical constriction forces and whether it would persist over time. To assess the sensitivity and specificity of the IRF3 and p65 antibodies, MDA-MB-231 cells were first treated with the cGAS agonist G3-YSD, what resulted in a 1.51-fold increase in cellular IRF3 and 1.59-fold increase in nuclear p65 (Fig. 6g). Nuclear translocation of p65 was not detected (Fig. 6g and Fig. S11), prompting us to quantify nuclear intensity values in our analysis evaluation.

The intensity levels of nuclear NF-κB (canonical and non-canonical) and cellular phospho-IRF3 were measured on the cells after they transited 5 µm constrictions and compared to their controls and expanded counterparts (Fig. 6a–c). Nuclear STAT1 and nuclear STAT3 were measured in cells that transited 5 µm constrictions and compared to their controls (Fig. 6e,f). MCF-10A was the cell line that had a larger fold-change increase in p65 (1.80-fold increase), RelB (1.47-fold increase), IRF3 (1.87-fold increase) and STAT1 (1.18-fold increase) intensity upon transit. MDA-MB-231 still had an increase in p65 (P < 0.0001), RelB (P < 0.0001), IRF3 (P < 0.0001), and STAT1 (P < 0.001) intensity signal, while fold-changes were smaller in LM2 cells, except for STAT3 (1.15-fold change). A comparison between STAT1 fold-activation induced by 5 µm constrictions or by interferon-α was included in Fig. S12. Only MDA-MB-231 and MCF-10A presented a persistent activation of some of these inflammatory markers in the expanded cells (Fig. 6h). Overall, these results suggest that constrictions forces can trigger the activation of the inflammatory pathways NF-κB and STAT and that these effects seem to be more pronounced in the cell line MCF-10A.

### Effects of mechanical constriction forces in transcriptomics

**Figure 7:**
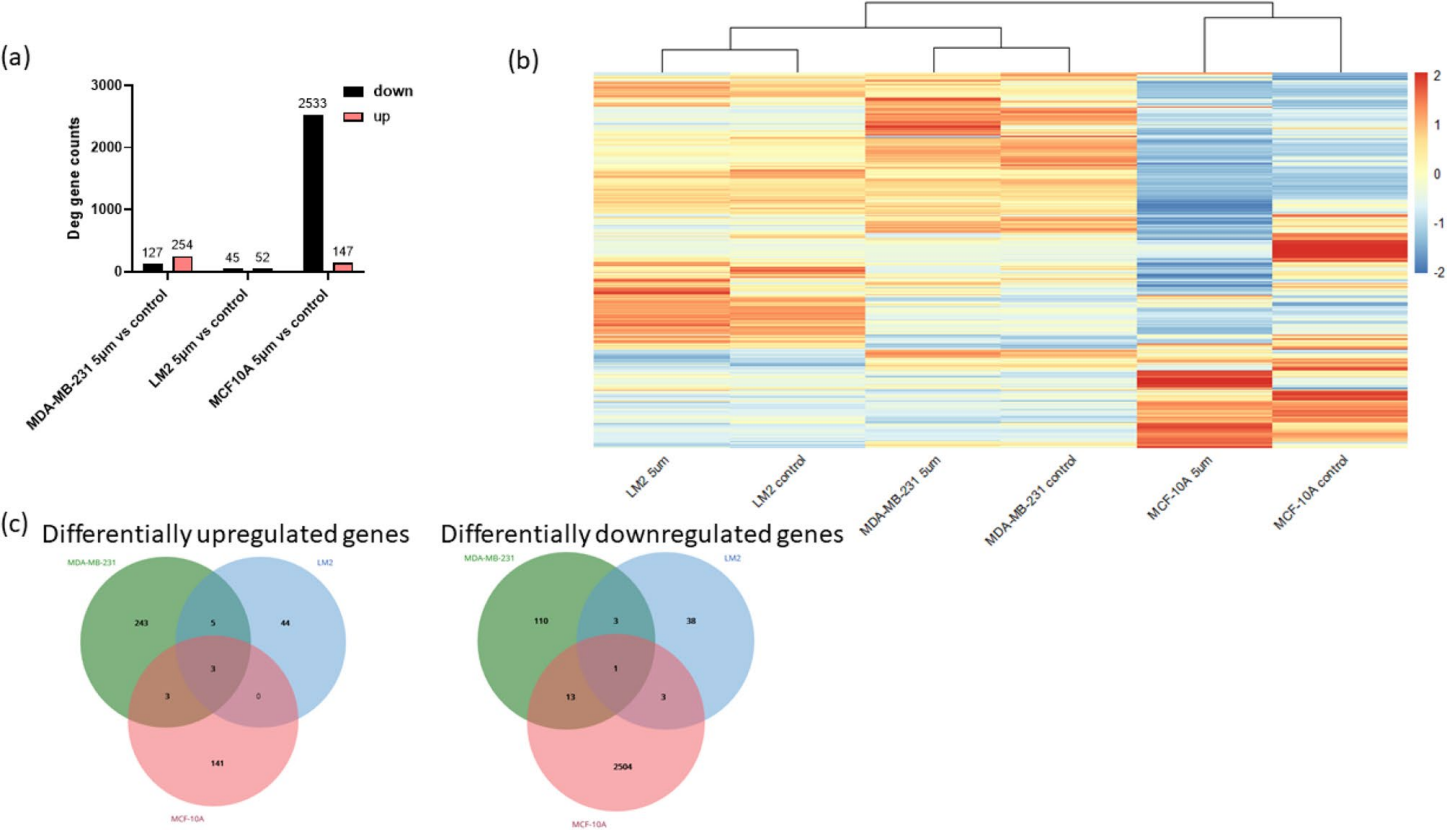
Effect of capillary constriction forces on RNA expression. Transcriptomics of MDA-MB-231, LM2 and MCF-10A cells after transiting 5 µm constrictions compared to cells that did not transit 5 µm constrictions (control). (**a**) Number of differentially downregulated and upregulated genes. (**b**) Heatmap of the differentially expressed genes with hierarchical clustering based on gene expression. (**c**) Venn diagram of the differentially expressed genes across cell lines after transiting 5 µm constrictions (left upregulated, right downregulated).

To further characterise the cell response to constricted transit through capillaries, we investigated how mRNA expression of MDA-MB-231, LM2 and MCF-10A cells that transited the constriction changed when compared to cells that did not experience constricted transit (control). For this aim, we performed mRNA extraction of MDA-MB-231, LM2 and MCF-10A cells that just transited 5 μm constrictions and their corresponding controls, followed by RNA sequencing and analysis. Differential expression analysis revealed that MCF-10A was the cell line with a larger number of differentially expressed genes (2680 genes), in which most of them were downregulated (2533), followed by MDA-MB-231 (381 genes differentially expressed) and LM2 (97 genes differentially expressed) (Fig. 7a). Hierarchical clustering of gene expression confirmed that MDA-MB-231 and LM2 transcripts were more similar to each other than to MCF-10A, even after transiting the constriction, which was expected as LM2 is a derivative of MDA-MB-231 (Fig. 7b). The differentially expressed genes after transit differed widely across cell lines, with only a few genes in common (Fig. 7c and Supplementary Tables 3 and 4), suggesting that constrictions trigger different transcriptional outcomes.

We next looked at the function of the genes that were differentially expressed (Table 1a). For the cell line MCF-10A, we observed downregulation of several cadherins (e.g. *PCDHA10, PCDHB10, PCDHB11*), collagen (*COL5A3, COL11A2, COL28A1* and *COL9A3*), fibronectin (*FNDC11*), integrins (*ITGAM *and* ITGB7*), keratins (e.g.* KRT4, KRT13, KRT8P46*) and the laminin *LAMB4*. Additionally, we detected downregulation of the senescence marker *TGFB2-AS1* and of different genes encoding proteins of the tumour suppressor p53, such as *PICART1, CFAP53* or *LINC-PINT*. Genes located in the nuclear envelope, such as nesprin-4 (*SYNE4*), involved in regulating nuclear migration and cell polarisation^43^, or the nucleoporins *HOXA7* and *NUP210L*, which have been previously reported to increase metastasis^44,45^, were also differentially expressed. Finally, different clusters of histones were found to be downregulated (e.g. *HIST1H2BG, HIST1H4H, HIST1H2AE*), except the histone *HIST2H2AC*, which was upregulated and has been reported to be highly expressed in breast cancers, promoting cell proliferation and EMT^46^. The dysregulation of the clusters of histones prompted us to evaluate the chromatin condensation state in cells that had transited constrictions compared to control and expanded cells by analysing nuclear Hoechst intensity levels, nuclear Hoechst SER and Gabor textures (Fig. S13). Our results suggest different states of chromatin compaction among cells that had transited constrictions, control and expanded cells, as these cell lines did not cluster together in the PCA space (Fig. S13a). In fact, hierarchical clustering using the same features suggested that chromatin features were more similar in MCF-10A and MDA-MB-231 that had transited constrictions (Fig. S13b).

**Table 1:** (a) Relevant genes dysregulated in the MCF-10A cell line after transiting 5 × 5 μm^2^ constrictions, (b) selection of relevant MCF-10A Gene Ontology processes.

(a)					
Gene	log2FoldChange	padj	Gene	log2FoldChange	padj
Cadherins			Senescence		
*PCDHA10*	− 7.47114	0.016408	*TGFB2-AS1*	− 5.06774	0.357877
*PCDHB10*	− 9.39833	0.027674	Keratins		
*PCDHB11*	− 8.55847	0.034324	*KRT4*	− 10.0303	0.002976
*PCDHB11*	− 8.55847	0.034324	*KRT13*	− 8.67278	0.004007
*PCDH12*	− 7.53895	0.040418	*KRT8P46*	− 7.67119	0.01318
*PCDHGA2*	− 6.87659	0.041356	*KRT12*	− 7.07637	0.05632
*CDHR3*	− 6.55311	0.062844	*KRT18P34*	− 6.87388	0.076218
*PCDHB15*	− 6.75782	0.085467	*KRT1*	− 8.81442	0.07647
*PCDHB5*	− 6.85576	0.087681	*KRT16P6*	− 6.32408	0.11227
*PCDHGA1*	− 7.40606	0.090732	*KRT222*	− 6.15079	0.146796
*CDH8*	− 6.48599	0.103136	*KRT19*	− 5.7107	0.256548
*PCDHB2*	− 5.38251	0.121237	*KRT39*	− 6.32116	0.309087
*PCDHB7*	− 7.24736	0.125076	*KRT8P39*	− 5.22499	0.32102
*PCDHB9*	− 6.67122	0.161215	*KRT16*	− 1.24469	0.323231
*PCDHGB6*	− 5.75916	0.176917	Apoptosis p53		
*CDHR5*	− 6.23747	0.194639	*PICART1*	− 7.90927	0.011045
*PCDHB6*	− 6.04864	0.216139	*AC007906.2*	− 7.43572	0.017185
*PCDHGB1*	− 5.68552	0.243059	*CFAP53*	− 7.85706	0.02394
*PCDHA2*	− 5.307	0.307133	*LINC-PINT*	− 6.02529	0.121237
*JCAD*	− 5.28127	0.328951	*TP53AIP1*	− 5.89098	0.140563
*PCDHGA11*	− 5.10467	0.344436	Nuclear envelope		
*PCDH15*	− 5.43668	0.357458	*SYNE4*	− 8.04737	0.00013
Collagen			*NUP210L*	6.528378	0.29522
*COL5A3*	− 8.69526	0.014689	*HOXA7*	23.47039	0.001048
*COL11A2*	− 6.56446	0.057927	Histones		
*COL28A1*	− 8.42792	0.12837	*HIST1H2BG*	− 7.68866	0.016882
*COL9A3*	− 5.92674	0.138418	*HIST1H4H*	− 7.79653	0.024001
Fibronectin			*HIST1H2BH*	− 6.81128	0.042034
*FNDC11*	− 5.32936	0.274683	*HIST4H4*	− 6.41946	0.070053
Laminin			*HIST1H2AI*	− 6.3104	0.082072
*LAMB4*	− 6.15708	0.102535	*HIST1H4J*	− 5.45589	0.263086
Integrins			*HIST2H2AC*	3.359096	0.344986
*ITGAM*	− 8.27012	0.07792			
*ITGB7*	− 6.96681	0.041166			

(b)					
Category	GOID	Description	GeneRatio	padj	
CC	GO:0031012	Extracellular matrix	60/1010	6.72E−08	
CC	GO:0005578	Proteinaceous extracellular matrix	51/1010	8.04E−08	
BP	GO:0007156	Homophilic cell adhesion via plasma membrane adhesion molecules	31/961	6.15E−06	
BP	GO:0098742	Cell–cell adhesion via plasma-membrane adhesion molecules	37/961	6.11E−05	
MF	GO:0005125	Cytokine activity	24/953	0.000767	
MF	GO:0022839	Ion gated channel activity	34/953	0.001045	
BP	GO:0031424	Keratinization	21/961	0.004352	
CC	GO:0032982	Myosin filament	6/1010	0.006529	
BP	GO:0043117	Positive regulation of vascular permeability	6/961	0.006764	
BP	GO:0016339	Calcium-dependent cell–cell adhesion via plasma membrane cell adhesion molecules	11/961	0.007996	
BP	GO:0006959	Humoral immune response	24/961	0.007996	
BP	GO:0006936	Muscle contraction	37/961	0.007996	
BP	GO:0050727	Regulation of inflammatory response	35/961	0.017093	
MF	GO:0004896	Cytokine receptor activity	12/953	0.031379	
BP	GO:0071219	Cellular response to molecule of bacterial origin	22/961	0.037557	
BP	GO:0046879	Hormone secretion	31/961	0.039811	
BP	GO:0043269	Regulation of ion transport	50/961	0.042732	
CC	GO:0005859	Muscle myosin complex	5/1010	0.0459	
BP	GO:0032609	Interferon-gamma production	14/961	0.04625	
BP	GO:0050900	Leukocyte migration	37/961	0.047404	
BP	GO:0043062	Extracellular structure organization	38/961	0.048152	

GO enrichment analysis in MCF-10A highlighted pathways involved in cell contractility (such as myosin filament, muscle contraction and muscle myosin complex). Pathways related to ECM, cell–cell adhesion and leukocyte migration, which are commonly activated in CTCs^47–49^, were also detected, together with pathways involved in inflammation, such as humoral immune response, cytokine activity or regulation of an inflammatory response (Table 1b).

Interestingly, pathways involved in EMT were not differentially regulated in the MDA-MB-231 and LM2 cell lines following constrictions. In fact, only five statistically significant GO pathways were detected for MDA-MB-231, whereas no statistically significant KEGGS or GO was detected for LM2 (Supplementary Enrichment). Notably, in MDA-MB-231, two of the enriched GO pathways were related to H3-K4 methylation. This suggests that constriction forces might still induce a regulation of chromatin and nuclear mechanics in MDA-MB-231 since H3-K4 methylation are euchromatic marks that have been linked with an increase of gene transcription^6^, cell stemness and tumorgenicity^50^ and with the regulation of nuclear stiffness in confined cells^51^.

Overall, most of the significant transcripts that were observed corresponded to MCF-10A, suggesting that cells with a more epithelial/non-malignant phenotype show more changes in gene expression upon constricted migration. The differential expression of the panel of genes and enrichment analysis that was detected are compatible with a transition towards a more mesenchymal phenotype in the MCF-10A cell line.

## Discussion

Because cancer is a much more treatable disease as long as cells remain localised in the primary tumour, understanding the drivers of metastatic dissemination is fundamental to target metastasis on its early stages. Since traversing capillary beds is one of the key steps during metastatic dissemination, we sought to investigate the effects that this transit has on CTCs. Due to the intrinsic difficulty of investigating this process in vivo, we developed a high-throughput microfluidic platform with capillary-like constrictions that recreated CTCs capillary transit under physiological fluid forces, while enabling the collection of a sufficient number of cells for downstream analysis.

Although the migration of cells through interstitial spaces is well documented^7–13^, the key differences between constricted migration and capillary transit described in the introduction make difficult to extrapolate the results that are known from constricted migration experiments. Moreover, differences in the microenvironmental conditions, such as the ECM and stromal cells encountered in solid tumours, which can promote cancer migration, or the higher exposure of CTCs to immune surveillance in capillary beds, which can be a survival limiting factor^5^, need to be considered. In our model, we observed that cells transiting capillaries experienced NE rupture only when traversing very narrow constrictions (5 × 5 µm^2^), similarly to what it is known from studies investigating constricted migration, which have reported that cells migrating through microfluidic channels of less than 4 × 5 µm^2^ cross-sections resulted in a tenfold increase of NE rupture events when compared to unconfined cells^13^. However, our results suggested that transit through capillaries led to more nuclear rupture events than constricted migration, since we observed a higher percentage of nuclear rupture in cells transiting 5 × 5 µm^2^ channels than the observed by Denais et al*.* in cells undergoing constricted migration through 4 × 5 µm^2^ constrictions^13^. We hypothesise that these differences might be a consequence of the faster time scales at which cells transit constrictions while being pushed by fluid forces, what might have given them less time to reorganise their cytoskeleton. Interestingly, despite the high percentage of NE rupture that both cancer and normal cells experienced, they remained viable, suggesting that both epithelial and mesenchymal phenotypes have mechanisms to overcome NE rupture. Another similarity we observed with cells undergoing constricted migration was a transient increase in lamin A/C folding and wrinkling, together with an increase in perinuclear and nuclear lamin A/C levels^10,24^. We believe this could be a mechanism adopted by the cell to stabilise the nucleus against the stress induced by the extreme deformation, as reported by Swift et al*.*^39^, or a consequence of NE rupture since it is known that increases in lamin A/C signal intensity in the nuclear ring are linked to lamin scars where NE rupture took place^13^. Conversely, recent evidence suggests that under constricted migration, cancer cells can decrease their nuclear stiffness^52,53^, but there is no indication yet of CTCs adopting this mechanism while transiting capillary beds. Our model suggests that this might be possible as we observed a persistent decrease of 13% and 16% in nuclear lamin A/C levels in the cell lines MDA-MB-231 and LM2, respectively, after expanding them for 2–5 passages after transit. A reduction in lamin A/C levels might be a mechanism adopted by cancer cells to decrease their nuclear stiffness and increase their nuclear deformability in order to increase cell migration and their metastatic potential^54–56^. Intriguingly, this decrease was not observed in the epithelial cell line MCF-10A, suggesting that this could be a mechanism more readily adopted by malignant cells.

Because tumour cells are known to be more plastic than non-malignant cells and can change their phenotype to overcome the different steps of the metastatic cascade^57^, we were interested in determining how a biophysical cue such as mechanical constriction forces, could alter the phenotype of both mesenchymal and epithelial cells and influence their metastatic potential. This prompted us to include in our study normal epithelial (MCF-10A) and mesenchymal cell lines with different metastatic potential (MDA-MB-231 and its lung-selective metastatic derivative LM2). Our results showed that both cancer and normal cell lines presented morphological changes after transiting capillaries, displayed by a nuclear and cytoplasmic area reduction. These results are consistent with both in vivo studies, showing that CTCs traversing lung capillaries lost cytoplasmic volume^58^, and with in vitro studies, in which a rapid mechanical compression resulted in cell volume loss^59,60^. We believe that the observed loss of cytoplasmic and nuclear area might be a consequence of the sudden accumulation of tension in the cell induced by mechanical compression. Strikingly, in our experiments the loss of cytoplasmic and nuclear area was not completely recovered over time in the metastatic cell lines, as their cytoplasm and nuclei area remained smaller than their controls after passaging the cells 2–5 times after capillary transit. This suggests that constriction forces might induce a persistent reduction in both cytoplasmic and nuclear area in the metastatic cell lines. Whether the loss of size might confer CTCs with an advantage to extravasate or migrate to another capillary bed with a more favourable soil, as smaller nuclei have been previously shown to improve the migration of cancer cells in vivo^61^, needs to be further investigated since there is no evidence yet of smaller cells being more metastatic^62^. In fact, a study evaluating single-cell morphological features in mouse models showed that breast cancer cell nuclei with a larger and rounder morphology were actually more metastatic than cells of smaller sizes^63^.

Little is known about how capillary constriction forces affect cell transcriptomics, partially because of the inherent difficulty of isolating CTCs after their transit through capillary beds. In this study we could overcome this limitation by using a high-throughput microfluidic platform that enabled the collection of a sufficient number of cells to perform RNA seq after experiencing capillary transit. Our results shed light not only on how capillary constriction forces affected gene expression but also on how cells with mesenchymal and epithelial phenotypes responded differently to this transit. This is relevant because CTCs have shown to acquire hybrid phenotypes during their metastatic dissemination to better adapt to different microenvironments^64^, suggesting that a change of phenotype could give an advantage to CTCs to overcome capillaries. Interestingly, we observed that epithelial phenotypes had more differentially expressed genes than mesenchymal phenotypes upon constricted transit and that these changes were compatible with EMT, suggesting that mechanical constrictions alone could prompt a transition into more malignant phenotypes. The downregulation of genes involved in extracellular matrix, cell adhesion and extracellular structure organization were in line with the few studies that performed RNA seq on cancer cells upon sequential rounds of constricted migration^24,65^. Golloshi et al*.* identified enriched pathways related to metastasis and downregulation of genes involved in cell–cell/matrix adhesion in MDA-MB-231^24^. Similarly, Fanfone et al. also observed in MDA-MB-231 cells enrichment of pathways involved in cell–matrix adhesion, ECM organisation and ECM disassembly^65^. It is remarkable that in these studies, sequential rounds of constricted migration could trigger downregulation of transcripts that were very similar to what we observed in our model after only one round of constricted transit.

Since the adaptation to biophysical cues of cancer cells in the different steps of cancer progression can lead to both a transient (e.g. the rapid cytoskeleton reorganisation cancer cells undergo to overcome the endothelial barrier during intravasation) and persistent response (e.g. the acquisition of a mesenchymal phenotype), we sought to investigate the duration of the effects that capillary constriction forces had on cancer and normal cells. This is relevant because a persistent response could pose an advantage for cancer cells to overcome the next steps of their metastatic spread, affecting cell fate. For this aim, we characterised cells immediately after having transited constrictions and after having expanded them for 2–5 passages post-transit. We observed an increase in proliferation that was maintained for 24 h after transiting capillaries in the normal breast cell line but not in the metastatic cell line, suggesting that cells with an epithelial phenotype might be more sensitive than tumorigenic mesenchymal cells to the effects of mechanical constriction forces, possibly because they have not been exposed to it before and have not adapted yet. Pfeifer et al. also observed an increase in proliferation on cells migrating through 8 µm pore constrictions, whereas opposite results were observed when cells transited smaller 3 µm-pore constrictions^10^. Further research in needed to understand the molecular mechanisms of the increase in proliferation we observed in our model in MCF-10A, but we propose that an increase in proliferation of cancer cells while arrested in capillaries could facilitate extravasation and metastasis as reported by Al-Mehdi et al*.,* who observed that metastasis was a result of the proliferation of cancer cells attached to the vascular endothelium rather than an extravasation of these cells in mice and rats models^66^.

Finally, in this study we tried to better understand the activation of the cGAS/STING pathway triggered by capillary transit. It is believed that in the cGAS/STING pathway the nature, intensity and duration of the initial trigger can lead to both chronic activation of the pathway, fostering metastatic dissemination, or to anticancer immunity if the initial trigger has a high intensity^67^. We were interested in finding out if transit through capillaries could induce a transient or a persistent activation of the cGAS/STING pathway and which downstream effectors would get activated. Our results suggest transient activation of the canonical NF-κB axis in MDA-MB-231 and MCF-10A, together with a remarkable activation of phosphorylated IRF3 in MCF-10A, which is known to mediate type 1 IFN response^67^. A stronger activation of the downstream effector STAT1 rather than STAT3, associated with a proinflammatory immune response and immune cell infiltration^68^, suggests that in our model capillary constrictions might trigger innate immune response activation. Interestingly, both MCF-10A and MDA-MB-231 showed persistent increase in the intensity signal of some of the cGAS/STING effectors that were evaluated. Specifically, MCF-10A had a sustained increase in dsDNA, cGAS, p65 and IRF3 signal, while MDA-MB-231 had a persistent increase in cGAS, STING, RelB and IRF3 signals, in contraposition to LM2 cells, which only had a transient increase in the signal of these proteins. Remarkably, in the LM2 cell line, the only persistent effects we detected were related to morphology (i.e., loss in nuclear and cytoplasm area) rather than to the activation of a pathway, suggesting that this metastatic cell line might be less responsive to mechanical constrictions.

Overall, our results support the idea that transit through capillary beds can induce transient and persistent effects on CTCs involved in the cGAS/STING activation, morphology and cytoskeleton reorganisation, together with differences in gene expression (suggesting a transition towards a more mesenchymal phenotype) that are more pronounced on epithelial phenotypes. These findings suggest that nuclear rupture alone might not be the only driver of these changes since both MDA-MB-231 and MCF-10A showed nuclear rupture but they experienced a very different response in terms of cGAS/STING activation, activation of inflammatory pathways and gene expression. Whether this is a consequence of adaptations made by the metastatic cell lines when they first encounter constricted transit in vivo since they were isolated from secondary tumours, or/and a result of the different mechanical properties across cell lines, such as cell viscoelasticity, needs to be further investigated.

## Materials and methods

### Microfluidic device design and fabrication

Photolithographic masks were designed using the Autodesk AutoCAD 2020 software platform. The master mould was composed of two different layers: constriction channels of 5 × 5 × 150 µm^3^ and 10 × 10 × 150 µm^3^ (width by height by length) were printed out on a first chrome mask, while a series of inlets and collector channels that converged in the centre of the chip were printed out on a second chrome mask (Micro Lithography Services, UK). Master moulds were fabricated following a standard photolithography protocol. To produce the first layer of the device, negative photoresist SU-8 GM1050 series (Gersteltec, Switzerland) was spin coated onto a 4-inch silicon wafer (Siegert Wafer, Germany) previously plasma treated for 45 s at 1450 rpm to obtain 5 µm-high features. For features of 10 µm-height, negative photoresist SU-8 GM1070 series (Gersteltec, Switzerland) was spin coated for 45 s at 2350 rpm. Wafers were baked for 2 min at 65 °C and for 20 min at 95 °C and then exposed to UV photolithography with a mask aligner (UV-KUB 3, KLOE, France) at 100% intensity for 4 s. A series of post-bakes of 2 min for 65 °C and 25 min for 95 °C were then applied to the wafer, followed by a SU-8 development step with propylene glycol methyl ether acetate (PGMEA, Sigma Aldrich, UK) for 20 s and a hard bake at 135 °C for 5 min. Wafers were then cleaned with acetone and IPA and the whole procedure was repeated to produce the second layer. SU-8 GM1070 was spin coated for 45 s at 3000 rpm to obtain a 30 µm-height layer, followed by a pre-baking step of 2 min at 65 °C for 20 min at 95 °C. A mask aligner (Karl Suss MA, Süss MicroTec, Germany) was used to align the two layers using aligning marks that were incorporated in the design of the mask, followed by a UV light exposure step with hard contact for 25 s. A post-baking step was applied by baking for 5 min at 65 °C, 35 min for 95 °C and 5 min for 65 °C. The wafer was developed for 2.5 min and hard baked. A surface profilometer (Dektak 6M Veeco, USA) was used to verify that the height was within the ± 10% of the target height. To produce the devices, Polydimethylsiloxane (PDMS, Dow Corning, UK) was mixed with its cross-linker at a 10:1 (w/w), degassed for 30 min using a vacuum desiccator, poured onto the silicon master moulds and cured overnight at 65 °C for 24 h. Cured PDMS was peeled off from the master mould and inlets and outlets were punched using a biopsy punch (Agar Scientific, UK) of 4 mm and 2 mm, respectively. PDMS devices were bonded to glass slides using a plasma cleaner (Harrick Scientific Products, USA) at 200 mmTor O_2_ at 45 W for 5 min and baked for 2 h at 100 °C.

### Cell culture

The panel of breast cancer cells that was investigated was composed of the following lines: MDA-MB-231 (purchased from ATCC), LM2 (a kind gift from Dr. Claus Jorgenson, Cancer Research UK Manchester Institute) and MCF-10A tagged for PCNA, which were a kind gift from Dr. Joerg Mansfeld^38^.

All cell lines except MCF-10A were cultured in cell culture medium StableCell™ DMEM—high glucose (Sigma-Aldrich) (4.5 g/L glucose, stable glutamine, and sodium bicarbonate) (Sigma-Aldrich), 10% heat-inactivated Fetal Bovine Serum (FBS) (Gibco) and 1% Penicillin/Streptomycin (Invitrogen). MCF-10A cells were obtained from ATCC and cultured in DMEM/F12 (31331, Gibco) supplemented with 5% horse serum (16050, Gibco), 10 μg/ml insulin (I-1882, Sigma), 20 ng/ml epidermal growth factor (E-9644, Sigma), 100 ng/ml cholera toxin (C-8052, Sigma), 500 ng/ml hydrocortisone (H-0888, Sigma), and 100 mg/ml penicillin/streptomycin (15070, Gibco). Cells cultures were maintained at 37 °C/5% CO_2_/4% O_2_ incubators. Cells were tested regularly for mycoplasma (e-Myco plus Mycoplasma PCR Detection Kit or iNtRON Biotechnology and MycoStrip 100, Invivogen).

Cells were passaged or collected for downstream analysis when they were at 80% confluency. For cell passaging or cell collection, growth media was aspirated and cells were washed with PBS. Cells were detached from culture flasks by incubating them with 0.25% trypsin–EDTA (Gibco) for 2 min, followed by neutralisation with 5 ml serum-containing media and centrifugation at 1200 rpm for 5 min. Supernatant was aspirated being careful of leaving the pellet intact and cells were resuspended in 2–3 ml of fresh media. Cell suspension was transferred to a new culture flask or loaded into the microfluidic devices.

### Generation of NLS-GFP tagged MDA-MB-231 and NLS-GFP tagged MCF-10A

A stable MDA-MB-231 cell line with an NLS-GFP signal was created using transfection. pCDH-NLS-copGFP-EF1-BlastiS was a gift from Jan Lammerding (Addgene plasmid #132772; http://n2t.net/addgene:132772; RRID: Addgene_132772). A glycerol stock was created from the bacterial stab following the Addgene protocol. Plasmid DNA was isolated and concentrated using a QIAGEN Plasmid Maxi Kit (QIAGEN) following manufacturers indications and DNA concentration was measured using a nanophotometer.

HEK-293T were used to produce viral supernatant using a Qiagen Effectene Transfection Reagent (301425, Effectene). Manufacturers protocol was followed. Briefly, HEK-293T cells were cultured to 40% confluency in a T75 in full DMEM media. The following day, a DNA-Effectene mixture including psPAX2 (Addgene, 4400 ng per reaction), MD2.G (Addgene, 1800 ng per reaction) and the NLS-GFP plasmid (5800 ng per reaction) was added to the HEK-293T cells. Cells were cultured overnight and media with Effectene changed for fresh media, with an extreme care to not peel cells off. HEK-293T cells were cultured for 48 h after infection to provide sufficient time for the HEKs to release the viral particles into the media. Conditioned media was then collected, filtered with 0.45 µm filters (Millipore) and added to a flask of MDA-MB-231 and MCF-10A cells that were seeded the day before. After 72 h incubation with the conditioned media, transfected cells were selected with Blasticidin (0.5 µg/ml). Cells positive for NLS-GFP were selected with Fluorescent-Activated Cell Sorting, plated in single wells of a 96-well plate and grown as single clones to ensure a homogeneous NLS-GFP signal.

### siRNA transfection

Stocks of 20 µM of siLMNA and siLMNB1 were prepared. Briefly, for T25 plates, siRNA reverse transfections were performed by adding 5.12 µl of siRNA to each well followed by 640 µl of Opti-MEM^®^ Reduced Serum Media. After 5 min incubation, 640 µl of mix containing Opti-MEM and RNAimax reagent in a 125:1 ratio was added to the tube. Tubes were incubated at room temperature for 20 min to allow formation of siRNA–RNAimax complexes. Cells (30,000 cells/ml) resuspended in 4 ml of fresh media, were added into a flask with the siRNA–RNAimax, resulting in a total volume of 5.28 ml per flask: 5.12 µl siRNA (final concentration of 20 nM), 640 µl Opti-MEM, 640 µl Opti-MEM and RNAimax mix (125:1) plus 4 ml of cells. Cells were incubated for 2 days, followed by downstream analysis.

### Transfections

#### cGAS agonist (G3-YSD)

MDA-MB-231 cells were plated in a 384 well plate the night before and treated with cGAS agonist G3-YSD (InvivoGen) overnight. cGAS agonist was transfected into the cells using the Lipofectamine™ 3000 Transfection Reagent (Thermo Fisher L3000001) and following manufacturers indications. A final concentration of 25 ng per well was achieved by diluting a stock concentration of 5 ng/µl in 5 µl, which was added to 45 µl of Optimem, reaching a total volume of 50 µl per well. Cells were cultured overnight.

#### Interferon-alfa

MDA-MB-231 cells were plated the night before. Interferon-alpha (Sigma-Aldrich IF007) was added at a final concentration of 200 U/ml and was prepared by diluting the stock concentration of 1 × 10^8^ U/ml in full media. The stock concentration was prepared by diluting it in a buffer of 0.1% bovine serum albumin (BSA). Cells were incubated with the compound for 30 min prior fixation.

### Microfluidic device preparation

Devices were handled under aseptic conditions and sterilised by exposing them to UV light for 30 min. Channels were flashed with ethanol 70%, followed by a wash with PBS and a coating with BSA 3% (w/v) (112018, Sigma-Aldrich) to prevent cell clogging. BSA was incubated for 3 h at room temperature and washed out with PBS. Prior use, chips were individually checked under inverted microscope to verify correct channel dimensions and that air bubbles were not introduced during the multiple washing steps.

### Capillary constriction experiment

A meter-length of Tygon tubing (T4103, Qosina) with an inner diameter of 0.76 mm and outer diameter of 2.29 mm was coupled to 10 ml polypropylene syringes using 21G needles (NeedlEZ, UK) and primed with PBS to prevent the introduction of air bubbles into the device. The other ending of the tubing was connected to the outlet of the device and plungers were removed from the syringe. The syringe was then lowered 7–33 cm below the height of the device to apply a negative hydrostatic pressure of 7–33 cmH_2_O. The PBS in the inlet was aspirated, leaving 10 µl to prevent bubbles entering the device and the cell suspension was prepared as described above at a concentration of 0.5 × 10^6^ cells/ml and was loaded into the inlet reservoir of the microfluidic devices. Devices were left running for 45 min–1 h under aseptic conditions and regularly checked under the microscope to ensure adequate functioning of the device.

Upon completion, the outlet tubing was carefully removed from the device and 5 µl of fresh media was introduced in the outlet and resuspended gently to collect the cells located there. This step was repeated three times to ensure all the cells were collected. 384 tissue culture treated plate (Perkin Elmers) were coated with 0.1% (w/v) fibronectin (F0895, Sigma-Aldrich) in PBS for 30 min at RT and let dry. Collected cells were placed in the plates and incubated at 37 °C/5% CO_2_/4% O_2_ prior fixation with 4% PFA (Thermo Fisher) for 10 min at 37 °C/5% CO_2_/4% O_2_.

For the imaging of NLS-GFP MDA-MB-231 cells and NLS-GFP MCF-10A transiting the constrictions, the microfluidic devices were operated as described above. Microfluidic devices were placed in a Nikon Eclipse Ti2 Inverted Microscope (Nikon, Japan) coupled to an incubated humidified chamber (Okolab, Italy) at 37 °C/5% CO_2._ The microscope was attached with a monochrome Nikon DS-Qi2 camera and LED illuminator for phase-contrast and fluorescent imaging. The filters used were the following: DAPI (excitation 358 nm/emission 461 nm), GFP (excitation 488 nm/emission 509 nm), and RFP (excitation 558 nm/emission 583 nm). Videos were taken at 0.4 frames per second.

To quantify nuclear rupture in cells transiting the constriction, the reporter NLS-GFP MDA-MB-231 and NLS-GFP MCF-10A cell lines were used. Hoechst 33342 (62249, Thermo Fisher) and Red Calcein-AM (5 μm, ThermoFisher) were used to segment nuclei and cell cytoplasm. Cells transiting through the constriction were tracked manually and nuclear rupture was considered to take place when GFP signal was detected in the cytoplasm. Those cells in which the GFP signal overlapped with Hoechst signal while they were transiting the constriction were considered to not experience nuclear rupture. The percentage of cells experiencing nuclear rupture was calculated from the total number of cells that were tracked transiting the constriction. Four different microfluidic devices were used for the quantification. Cell velocity was calculated by individually tracking the time required for cells to transverse the constriction from the point the nucleus first encountered the constriction until it exited it.

### Immunofluorescence staining

Fixed cells with 4% FDA methanol-free (ThermoFisher Scientific, 28908) were washed with PBS and incubated with 40 µl of 0.2% Triton-X in PBS (w/v) solution for 10 min. Wells were washed with PBS and 40 µl of 2% BSA (w/v) was added and incubated for 1 h. All the steps were performed at RT. PBS was aspirated and the antibody solution mix was made up with 0.5% BSA, 0.01% Triton-X and PBS. Primary antibodies were added at the required concentration (Supplementary Table 2) and incubated overnight at 4 °C. Wells were washed with PBS and the secondary antibodies were added at a concentration of 1:500 diluted in the antibody solution mix and incubated for 2 h at RT. Wells were washed with PBS and Hoechst 33342 (62249, Thermo Fisher) was added at a concentration of 1:1000 in PBS and incubated for 10 min. Plates were washed with PBS and stored at 4 °C. Imaging was performed using an Opera high-throughput microscope (PerkinElmer) or Opera Phenix high-throughput microscope (PerkinElmer) with a 40× water lens. Lasers and their corresponding filters used were: 405 (450/50), 561 (600/40), 488 (540/751), 640 (690/50).

### Cell viability

Upon transiting the constriction, cells were collected and plated at the same cell densities in three different wells of a 96 well plate for the three different time points that were evaluated (1.5 h, 6 h and 6 days). Green Calcein-AM (5 μM, ThermoFisher), propidium iodide (PI) (1 μg/ml, ThermoFisher) and Hoechst 33342 were dissolved in PBS, added to the well and incubated for 20 min prior imaging. A different well was used per time point to prevent dye toxicity affecting cell viability. Positive controls for PI (dead cells) were added by incubating the cells for 15 min with Triton-X 0.2%. Wells were imaged with Nikon Eclipse Ti2 Inverted Microscope (Nikon, Japan) and quantification was performed with the NISElement AR 5.30 software.

### SEM images

SEM images were taken with a FIB-SEM Tescan Lyra3 XMU (Tescan, Czech Republic) at the Hamlyn Centre (Imperial College London) with acceleration voltages of 1 keV.

### Cell synchronisation

Proliferation activity in the cell line MDA-MB-231 and MCF-10A cell lines was evaluated in different phases of the cell cycle: G1/early S, G0 and non-synchronised cells. To synchronise cells at the G1/S phase, cells were seeded the day before in a T25 flask. 2.5 mM Thymidine (Sigma-Aldrich, T9250) was dissolved in 4 ml of full DMEM and added to cells after aspirating the media. Cells were cultured for 24 h, washed with PBS, and then cultured in fresh DMEM for 8 h, to which 10 µM BrdU thymidine analog labelling solution BrdU (ab142567) was added to validate the efficiency of the synchronisation. Cells were washed and incubated in 2.5 mM Thymidine dissolved in fresh media for 16 h, following the protocol published by Li et al.^69^. To synchronise cells in G0, cells were serum starved by culturing them for 24 h in DMEM without FBS. Non synchronised cells were cultured in full media. 10 µM BrdU thymidine analog labelling solution was added to the media and cells were cultured for 24 h. Media was changed at the same time points at which thymidine synchronised cells was replaced. Once cells were synchronised, they were trypsinised, resuspended in fresh media and fed into the microfluidic devices following the approach described in the section “Capillary constriction experiment”. Controls for each condition (thymidine synchronised, serum starved and non-synchronised cells) were cells that did not circulate through the constriction. Cells were fixed at 3 different time points: 30 min after transiting the constriction, 2 h and 24 h to provide sufficient time for the cells to transition to the next state of the cell cycle. 30 min and 2 h time points were chosen to determine if cells in G0 would transition into a proliferative state. 24 h time point was selected to determine if cells get arrested in the long term after transiting through the constriction. Cells were fixed in FDA 4%. Ki67 (ab16667) antibody was added into the staining solution, prepared as described in the immunofluorescence staining section. Nuclear average intensity was quantified.

### Cell cycle analysis using FACS

Cell cycle profiling using PI staining was performed according to standard procedures. Briefly, double thymidine block arrest at G1/S, serum starved at G0 and non-synchronised cells were harvested and washed once in cold PBS. Prior to staining, cells were fixed with cold 70% ethanol for 30 min, which was added drop-wise to the cell pellet. Cells were washed in PBS and incubated in 5 µg/ml PI and 25 µg/ml RNAse A at 37 °C for 30 min. FACS analysis was performed on BD LSR II Analyzer station. At least 10,000 cells per condition were gated by duplicate for each condition. Data were obtained using the FlowJo cytometry package using raw cytometry data. The Watson Pragmatic algorithm of the cell cycle platform was used for the fitting.

### Invasion assay

The invasion assay was performed with ECM gel from Engelbreth-Holm-Swarm murine sarcoma (Sigma-Aldrich, E6909). Ultra low attachment plates ULA (Greiner Bio-one, 650979) were used for the production of spheroids following the protocol from Pascual-Vargas et al.^70^. DMEM 5× was prepared using 12.5 g DMEM powder, 25 ml of 1 M HEPES pH 7.5, 5 g NaHCO_3_, H_2_O up to 250 ml and was filtered under sterile conditions before aliquoting and freezing it at − 20 °C. From the 5× concentrated aliquots, final aliquots of 2× were obtained by diluting the 5× stock in sterile H_2_O and adding 20% FBS. Cells were plated at a density of 5 × 10^4^ cells/ml diluted in 100 µl media per well. Cells suspension from cells going through the constriction was filtered using a tube with cell strainer cap (Fischer Scientific, 10585801) to remove PDMS debris from the microfluidics device. 2.5 µl ECM was added per well to promote spheroid formation. Spheroids were cultured for 48 h and after spheroid formation, 80 µl of media was removed and replaced with 80 µl 100% ECM. After letting collagen polymerise at 37 °C for 2 h, 100 µl media 2× concentrated was added on top and changed every day. Spheroids were imaged every day for 7 days using brightfield in a Nikon Eclipse Ti2 Inverted Microscope (Nikon, Japan). Cell area was measured using the NISElement AR 5.30 software. The percentage of increase of area was calculated with respect to cell area from day 3 after adding ECM in MDA-MB-231 and for LM2 and MCF-10A with respect to day 2 after adding ECM. These were the days at which spheroids stopped compacting and an increase in area was observed. Protrusions were counted manually.

### RNA extraction

MDA-MB-231, LM2 and MCF-10A cells were collected after transiting the microfluidic devices. To ensure sufficient cells were collected, between 16 to 20 chips were run in parallel per experiment. Cells suspensions were counted, and concentrations ranged from 2.7 to 8.5 × 10^5^ cells/ml, diluted in 200 µl of media. Cell concentration and number of microfluidic chips used per experiment are described for each cell line in Supplementary Table 1. Once cells were collected, they were centrifuged to obtain a cell pellet and RNA and DNA (not sequenced in this experiment) were extracted using an All prep DNA/RNA Mini Kit (Qiagen). RNA concentrations were measured using a NanoPhotometer N50 (Implen, USA) and samples kept at − 80 °C until they were sequenced. For each cell line, each experiment was repeated twice to obtain biological replicates.

### RNA sequencing

Library preparation and data analysis was performed by Novogene. Briefly, RNA samples were subjected to QC, mRNA library preparations with poly A enrichment were done and samples were sequenced with Illumina Sequencing PE150 (12G raw data per sample, 40M reads), followed by bioinformatics analysis.

### Library construction, quality control and sequencing

Messenger RNA was purified from total RNA using poly-T oligo-attached magnetic beads. After fragmentation, the first strand cDNA was synthesised using random hexamer primers, followed by the second strand cDNA synthesis using either dUTP for directional library or dTTP for non-directional library. For the non-directional library, it was ready after end repair, A-tailing, adapter ligation, size selection, amplification, and purification. For the directional library, it was ready after end repair, A-tailing, adapter ligation, size selection, USER enzyme digestion, amplification, and purification. The library was checked with Qubit and real-time PCR for quantification and bioanalyzer for size distribution detection. Quantified libraries were pooled and sequenced on Illumina platforms, according to effective library concentration and data amount.

### Clustering and sequencing

The clustering of the index-coded samples was performed according to the manufacturer’s instructions. After cluster generation, the library preparations were sequenced on an Illumina platform and paired-end reads were generated.

### Data analysis

#### Quality control

Raw data (raw reads) of fastq format were firstly processed through fastp software. In this step, clean data (clean reads) were obtained by removing reads containing adapter, reads containing ploy-N and low quality reads from raw data. At the same time, Q20, Q30 and GC content of the clean data were calculated. All the downstream analyses were based on the clean data with high quality.

### Reads mapping to the reference genome

Reference genome and gene model annotation files were downloaded from genome website directly. Index of the reference genome was built using Hisat2 v2.0.5 and paired-end clean 2 reads were aligned to the reference genome using Hisat2 v2.0.5. We selected Hisat2 as the mapping tool for that Hisat2 can generate a database of splice junctions based on the gene model annotation file and thus a better mapping result than other non-splice mapping tools.

### Quantification of gene expression level

featureCounts v1.5.0-p3 was used to count the reads numbers mapped to each gene. Then, FPKM (Fragments Per Kilobase of transcript sequence per Millions base pairs sequenced) of each gene was calculated based on the length of the gene and reads count mapped to this gene. FPKM considers the effect of sequencing depth and gene length for the reads count at the same time.

### Differential expression analysis

Differential expression analysis of two conditions/groups (two biological replicates per condition) was performed using the DESeq2 R package (1.20.0). DESeq2 provide statistical routines for determining differential expression in digital gene expression data using a model based on the negative binomial distribution. The resulting P-values were adjusted using the Benjamini and Hochberg’s approach for controlling the false discovery rate. Genes with an adjusted P-value < = 0.05 found by DESeq2 were assigned as differentially expressed.

### Enrichment analysis of differentially expressed genes

Gene Ontology (GO) enrichment analysis of differentially expressed genes was implemented by the clusterProfiler R package, in which gene length bias was corrected. GO terms with corrected P value less than 0.05 were considered significantly enriched by differential expressed genes. ClusterProfiler R package was used to test the statistical enrichment of differential expression genes in KEGG pathways^71^.

### Gene set enrichment analysis

Genes were ranked according to the degree of differential expression in the two samples, and then the predefined Gene Set were tested to see if they were enriched at the top or bottom of the list. Gene set enrichment analysis can include subtle expression changes. A local version of the GSEA analysis tool http://www.broadinstitute.org/gsea/index.jsp was used. GO and KEGG data sets were used for GSEA independently.

### Image analysis

Immunofluorescence images were analysed using the Columbus and Harmony High-Content Analysis software (PerkinElmer) unless described otherwise. For all the confocal images, a maximum intensity projection of all the planes (2–3 planes were taken in each image with a step size of 1.5 µm, unless otherwise specified) was produced prior segmentation. Cell nuclei were segmented using Hoechst. The cytoplasmic region was defined as the ring region around the nucleus (defined as 25% outer border, 47% inner border) and it was used for the calculation of nuclear translocation (nuclear/cytoplasmic region intensity) as a normalisation of the cytoplasmic size (Supplementary Fig. S14). Cytoplasm was segmented with Phalloidin or Tubulin, depending on the experiment. Perinuclear area was defined as the ring surrounding the nucleus (defined as 45% outer border and 55% inner border).

### Statistical analysis

Statistical analyses were performed using Prism 9 (GraphPad). Statistical test for significant differences among groups of a single variable was carried out using ANOVA, followed by a Tukey multiple comparison test. When only two groups were compared, a two-tailed *t* test was applied. Hierarchical clustering and heat maps were made with Morpheus Broad Institute Software and with the R software after applying a Z-score transformation to the data. PCA analysis was performed using Statistics Kingdom (2017). *Multiple Linear Regression Calculator* (web application].

### Supplementary Information

Supplementary Information 1.

Supplementary Information 2.

## Data Availability

The data discussed in this publication have been deposited in NCBI's Gene Expression Omnibus^72^ and are accessible through GEO Series accession number GSE246824 (https://www.ncbi.nlm.nih.gov/geo/query/acc.cgi?acc=GSE246824).
