# Supplementary material for: Nuclear rupture induced by capillary constriction forces promotes differential effects on metastatic and normal breast cells
Source: Sci Rep. 2024 Jun 26;14:14793. doi: 10.1038/s41598-024-64733-x (PMC11208511; doi:10.1038/s41598-024-64733-x)
Supplement: Supplementary file 2 — Supplementary Information 2. [file 41598_2024_64733_MOESM2_ESM.docx]

Supplementary Figures


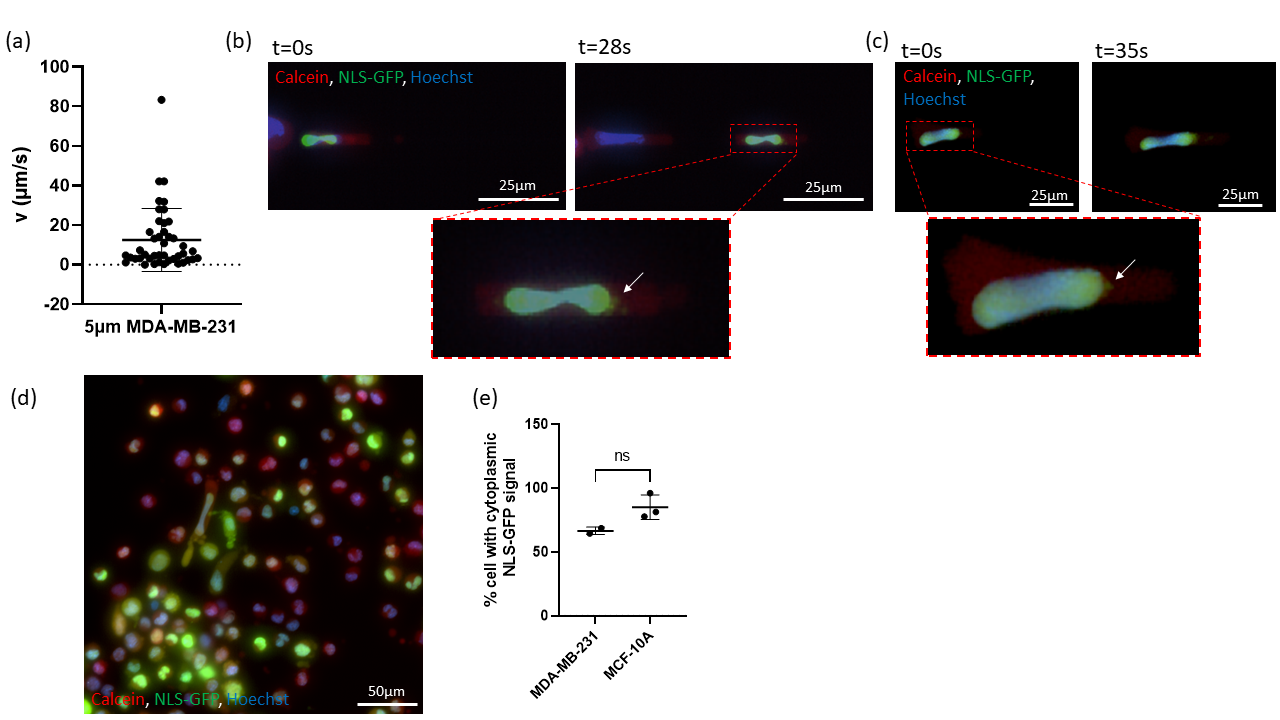


**Fig.S1. Characterisation of capillary transit and NE rupture events.** (a) Velocity (µm/s) of MDA-MB-231 cells transiting 5x5µm^2^ constrictions. Each dot represents a cell. (b) Live-imaging of a NLS-GFP MDA-MB-231 cell experiencing NE rupture 28s after entering a 5x5μm^2^ constriction and of a (c) NLS-GFP MDA-MB-231 cell experiencing NE rupture at the entrance point of a 5x5μm^2^ constriction. White arrows highlight the point of nuclear rupture. (d) Fluorescence image of the outlet area of a microfluidic device comprising NLS-GFP MDA-MB-231 cells 30min after transiting 5x5µm^2^ constrictions. (e) Percentage of MDA-MB-231 (n=129) and MCF-10A (n=71) cells displaying cytoplasmic NLS-GFP signal 30min post transit. Each dot corresponds to a microfluidic chip replicate. Comparisons among groups were performed by unpaired t test, two-tailed (t=46.48, DF=6, ∗p < 0.05,∗∗p < 0.01,∗∗∗p < 0.001,∗∗∗∗p < 0.0001).


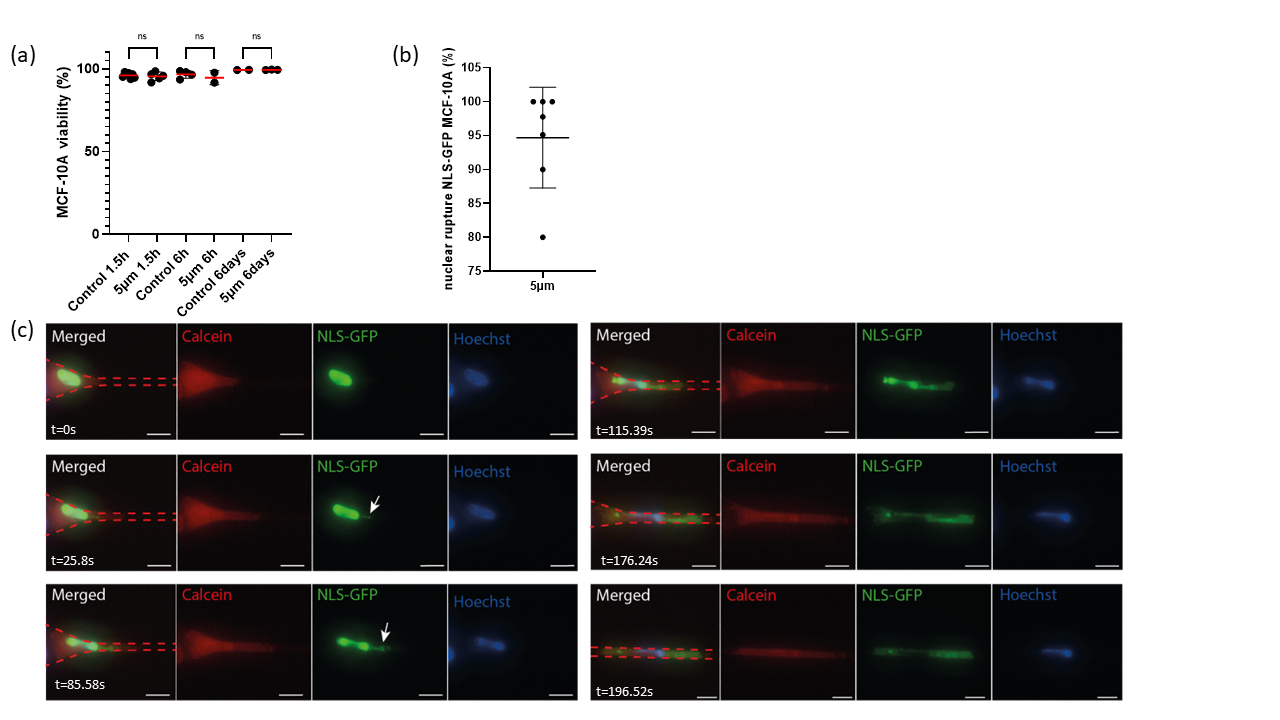
**Fig. S2.** **NLS-GFP MCF-10A cells transitioning through 5x5μm^2^ constrictions remained viable and experienced nuclear rupture**. (a) Cell viability (%) of MCF-10A cells 1.5h, 6h and 6 days after transiting 5x5μm^2^ constrictions compared to cells that did not transit the constriction. Each dot represents a biological replicate, including at least 100 cells per replicate. Comparisons among groups were performed by one-way ANOVA (Tukey multiple comparison test; ∗p < 0.05,∗∗p < 0.01,∗∗∗p < 0.001,∗∗∗∗p < 0.0001). (b) Percentage of NLS-GFP MCF-10A experiencing rupture while transiting 5x5μm^2^ constrictions. Each dot represents a biological replicate. A total of 149 cells were quantified. (c) Live-imaging of a NLS-GFP MCF-10A cell transiting a 5x5μm^2^ constriction. Cell nuclei was stained with Hoechst and cytoplasm was stained with Calcein Red-AM. White arrow indicates the moment in which first evidence of nuclear rupture takes place. Channel is highlighted with a red dashed line. Scale bar: 20µm.


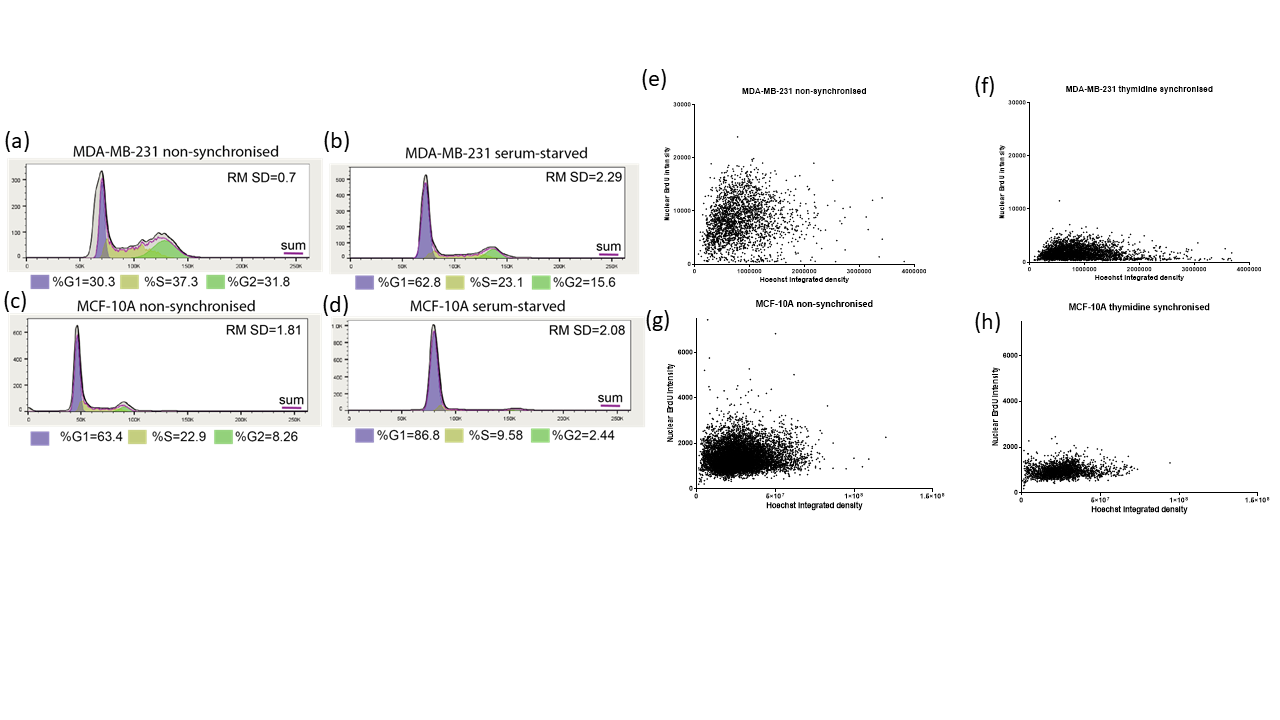


**Fig S3. Profiling of cell cycle in thymidine synchronised cells, serum starved and non-synchronised cells.** Profiling of cell cycle by FACS by using PI DNA staining: Percentage of MDA-MB-231 in G1, S and G2/M phase in an asynchronous population (a) and in a serum-starved population (b). Percentage of MCF-10A in G1, S and G2/M phase in an asynchronous population (c) and in a serum-starved population (d). Root-mean-square deviation (RM SD) from the model fitting is presented for each condition. Profiling of cell cycle by plotting Hoechst integrated density and nuclear BrdU in an asynchronous MDA-MB-231 population (e) and in a MDA-MB-231 population arrested by double thymidine block (f). Profiling of cell cycle by plotting Hoechst integrated density and nuclear BrdU in an asynchronous MCF-10A population (g) and in a MCF-10A population arrested by double thymidine block (h). Each dot represents a cell.

**
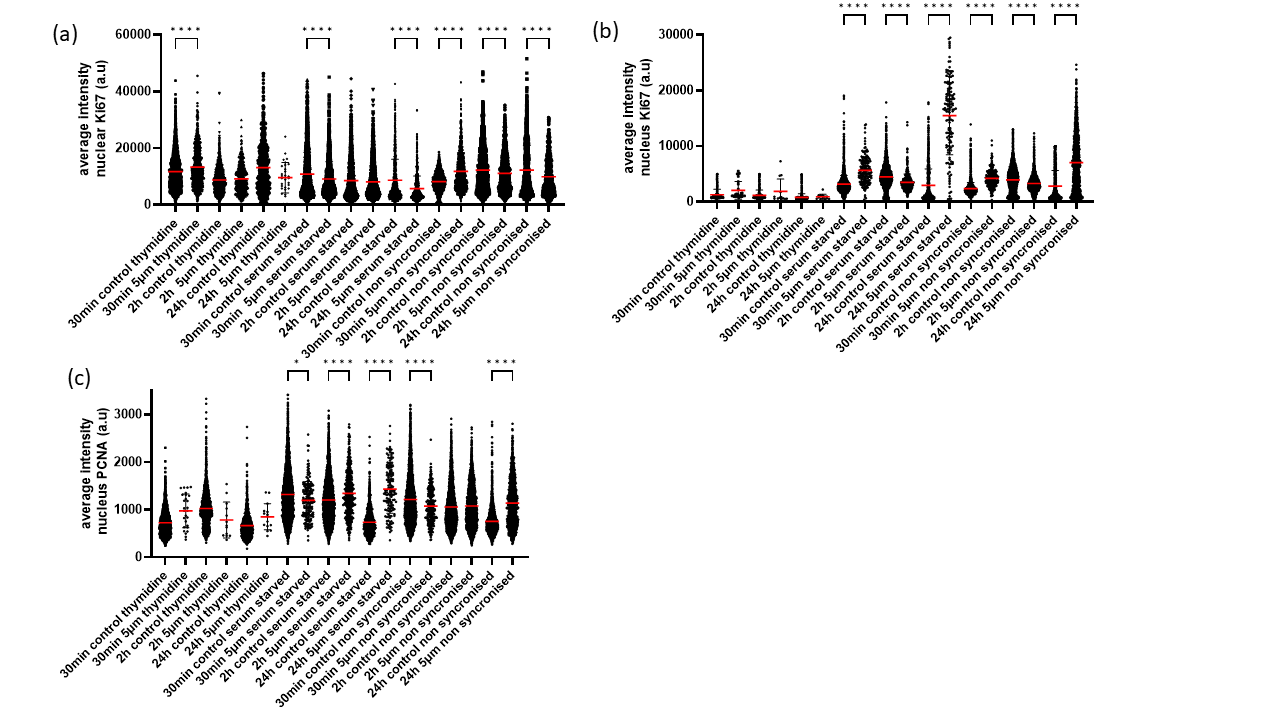
Fig. S4. Single cells results of Ki67 and PCNA signal**. Average intensity of nuclear Ki67 in MDA-MB-231 (a), average intensity of nuclear Ki67 in MCF-10A cells (b), and average intensity of nuclear PCNA in MCF-10A cells (c) 30min, 2h and 24h after transiting constrictions with respect to cells that did not transit the constriction. Cells where synchronised in two different phases of the cell cycle: G1/early S (thymidine synchronised) and G0 (serum starved for 24h) and compared to non-synchronised cells. Each dot represents a cell. For each group, means are highlighted in red. Within each cell line, comparisons among groups were performed by one-way ANOVA (Tukey multiple comparison test; ∗p < 0.05,∗∗p < 0.01,∗∗∗p < 0.001,∗∗∗∗p < 0.0001).


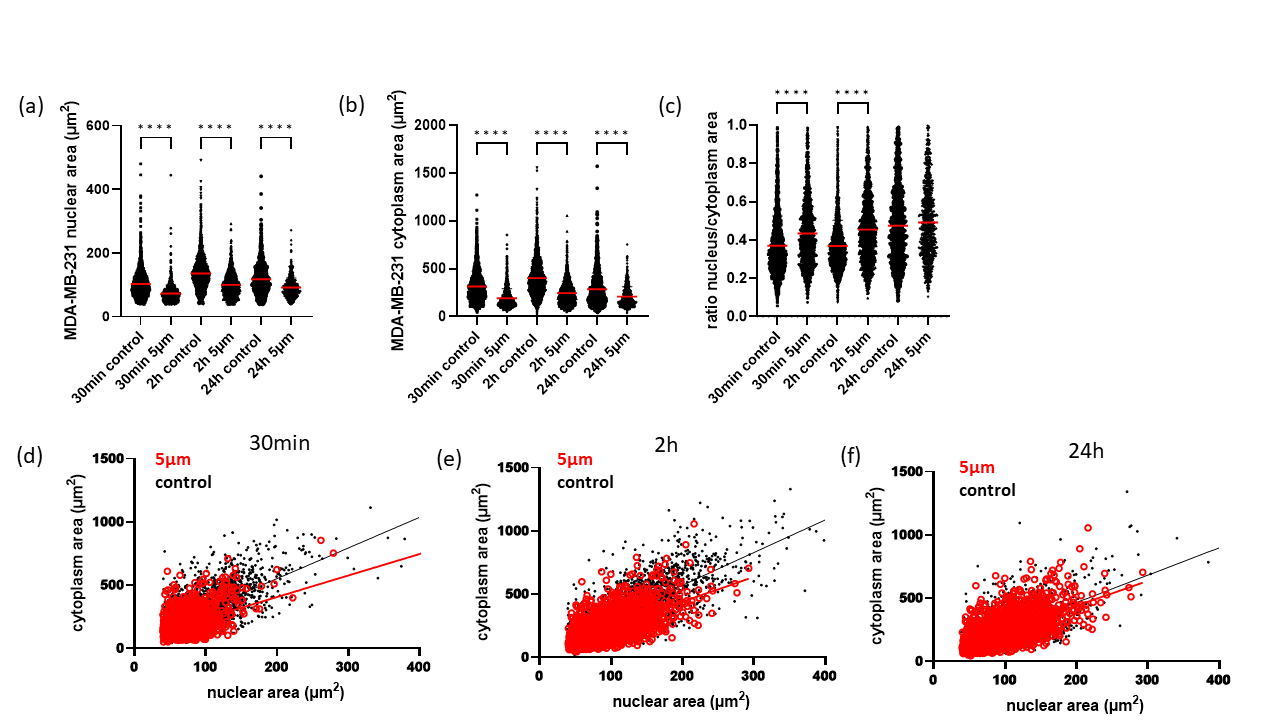


**Fig. S5. MDA-MB-231 nuclear and cytoplasmic area 30min, 2h and 24h after transiting 5µm constrictions compared to controls.** (a) MDA-MB-231 nuclear area (µm^2^) 30min, 2h and 24h after cells transited the 5µm constriction, (b) MDA-MB-231 cytoplasm area (µm^2^) 30min, 2h and 24h after cells transited the 5µm constriction, (c) ratio of nuclear/cytoplasm area 30min, 2h and 24h after cells transited the 5µm constriction. Within each cell line, comparisons among groups were performed by one-way ANOVA (Tukey multiple comparison test; ∗p < 0.05,∗∗p < 0.01,∗∗∗p < 0.001,∗∗∗∗p < 0.0001). (d) Correlation between nuclear and cytoplasm area (µm^2^) 30min after cells transited 5µm constrictions compared to control, (e) correlation between nuclear and cytoplasm area (µm^2^) 2h after cells transited 5µm constrictions compared to control, (f) correlation between nuclear and cytoplasm area (µm^2^) 24h after cells transited 5µm constrictions compared to control. Simple linear regressions for each dataset were plotted. In all the graphs, each dot represents a single cell.


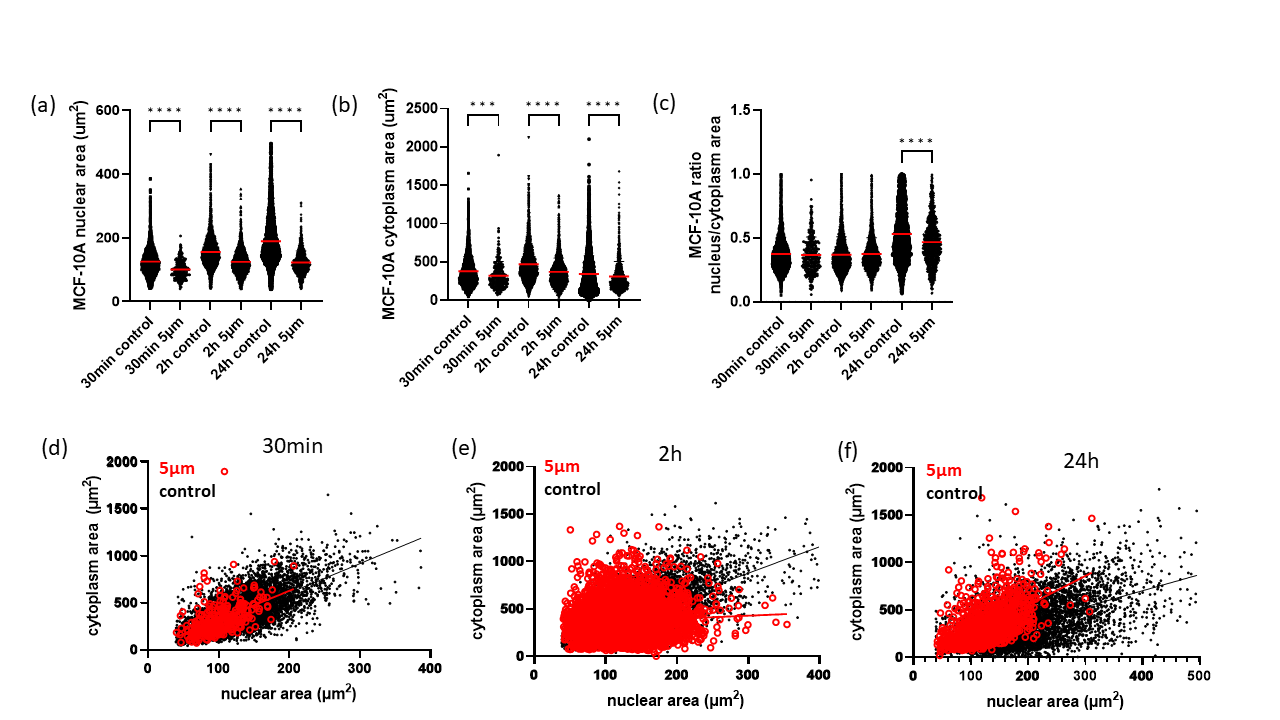


**Fig. S6. MCF-10A nuclear and cytoplasmic area 30min, 2h and 24h after transiting 5µm constrictions compared to controls.** (a) MCF-10A nuclear area (µm^2^) 30min, 2h and 24h after cells transited the 5µm constriction, (b) MCF-10A cytoplasm area (µm^2^) 30min, 2h and 24h after cells transited the 5µm constriction, (c) ratio of nuclear/cytoplasm area 30min, 2h and 24h after cells transited the 5µm constriction. Within each cell line, comparisons among groups were performed by one-way ANOVA (Tukey multiple comparison test; ∗p < 0.05,∗∗p < 0.01,∗∗∗p < 0.001,∗∗∗∗p < 0.0001). (d) Correlation between nuclear and cytoplasm area (µm^2^) 30min after cells transited 5µm constrictions compared to control, (e) correlation between nuclear and cytoplasm area (µm^2^) 2h after cells transited 5µm constrictions compared to control, (f) correlation between nuclear and cytoplasm area (µm^2^) 24h after cells transited 5µm constrictions compared to control. Simple linear regressions for each dataset were plotted. In all the graphs, each dot represents a single cell.


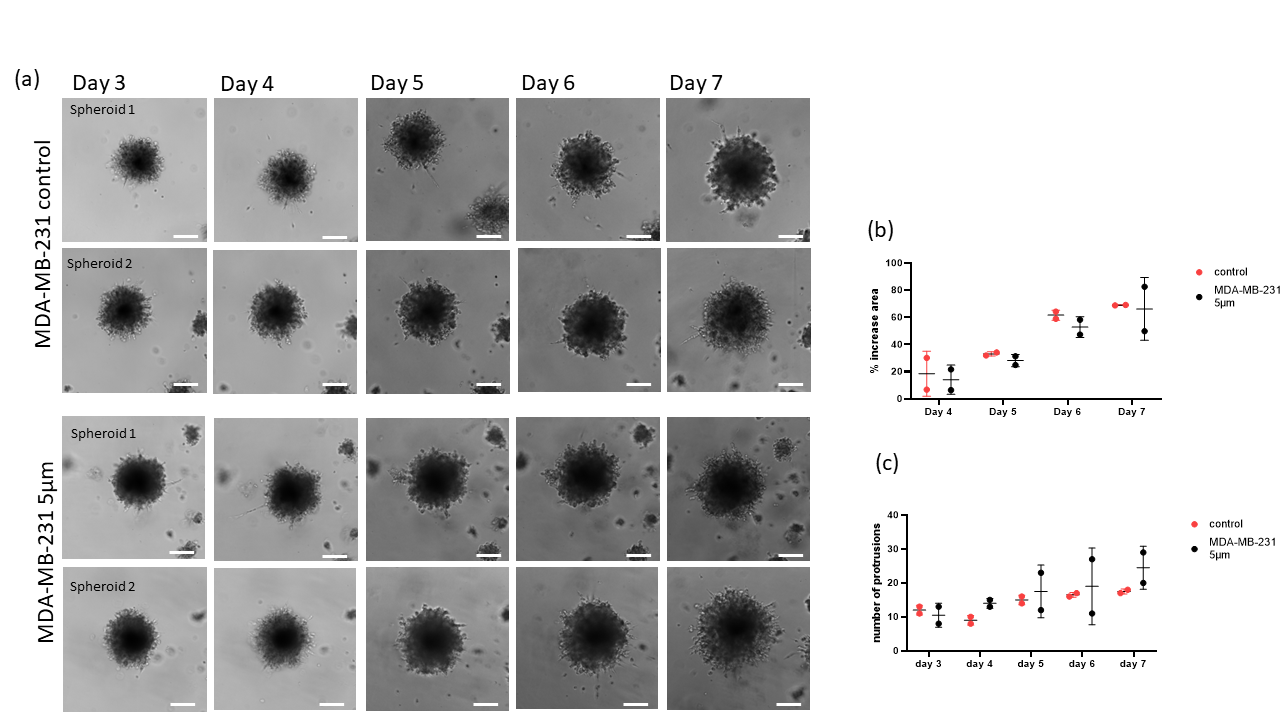


**Fig. S7**. **Invasion rates of spheroids composed of MDA-MB-231 cells that have transited the constriction compared to spheroids composed of cells that have not transited it (control).** (a) Brightfield images of the spheroids. Scale bar: 200µm. (b) Increase of spheroid area (%) on day 4, 5, 6 and 7 with respect to spheroid area during day 3, which is the time point at which spheroids stopped compacting. (c) Quantification of the number of protrusions in the spheroids. Each dot represents a spheroid.


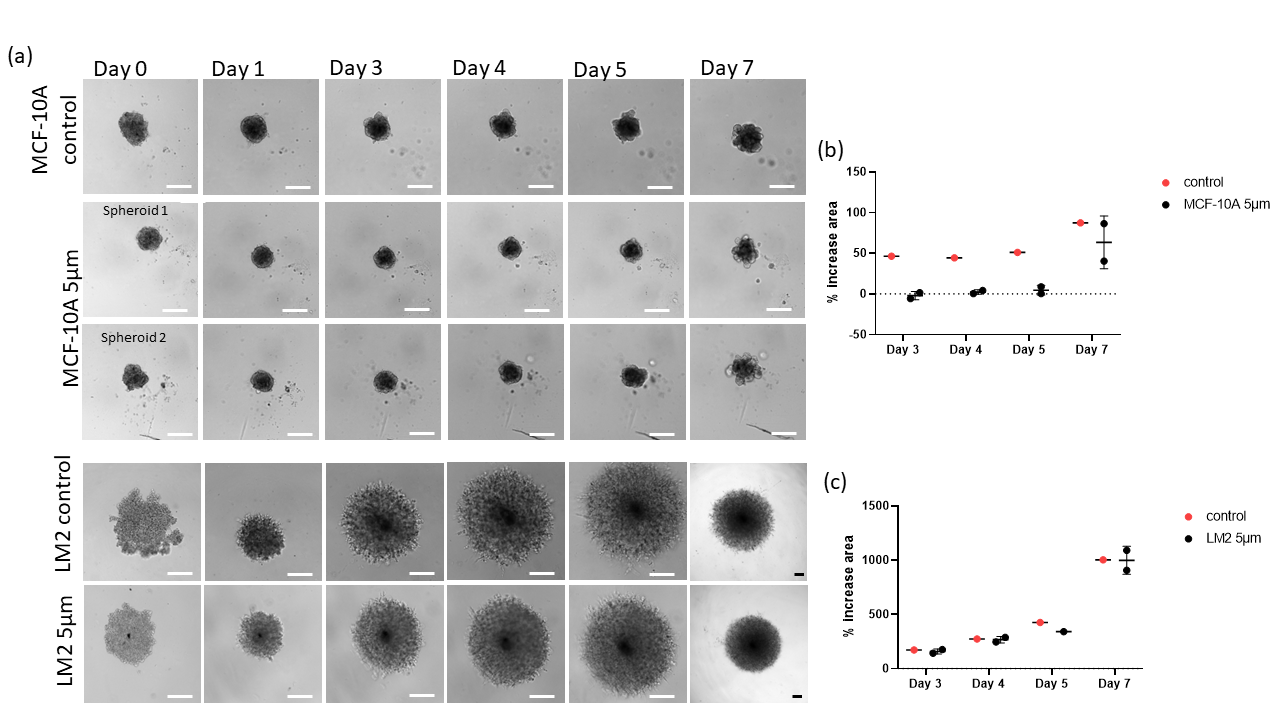


**Fig. S8. Invasion rates of spheroids composed of MCF-10A and LM2 cells that have transited the constriction compared to spheroids composed of the corresponding control cell.** (a) Brightfield images of the spheroids. Scale bar: 200µm. Increase of spheroid area (%) of MCF-10A (b) and LM2 (c) on day 3, 4, 5 and 7 with respect to spheroid area during day 2, which is the time point at which spheroids stopped compacting. Each dot represents a spheroid.


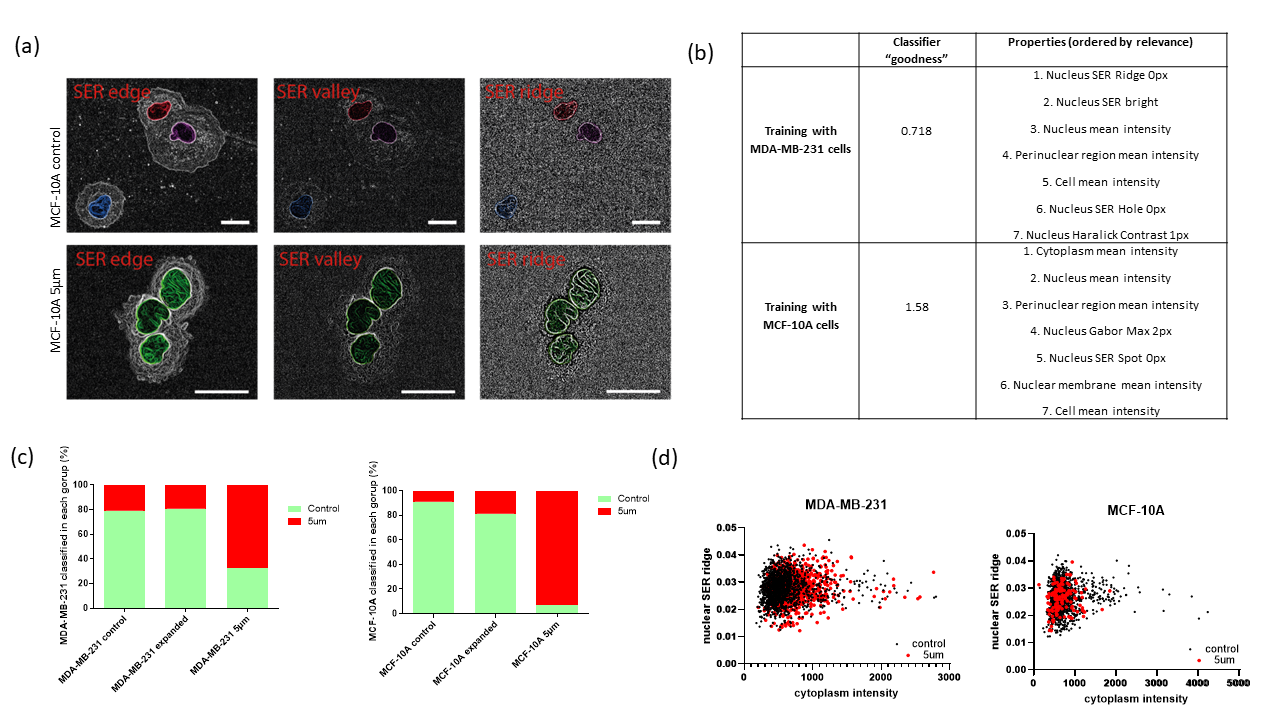


**Fig. S9**. **Texture analysis of nuclear intensity of lamin A/C.** (a) SER edge, SER valley and SER ridge of MCF-10A cells after transiting 5µm constrictions and control. Scale bar: 20µm. (b) Summary of a linear classifier manually trained using only lamin A/C features (intensity and texture). The classifier was trained separately for MDA-MB-231 and MCF-10A cells. (c) Percentage of MDA-MB-231 cells (left) and MCF-10A cells (right) that were assigned to the group constriction and the group control by the linear classifier. (d) Plot of cytoplasm mean intensity and nuclear SER ridge values of MDA-MB-231 (left) and MCF-10A (right).


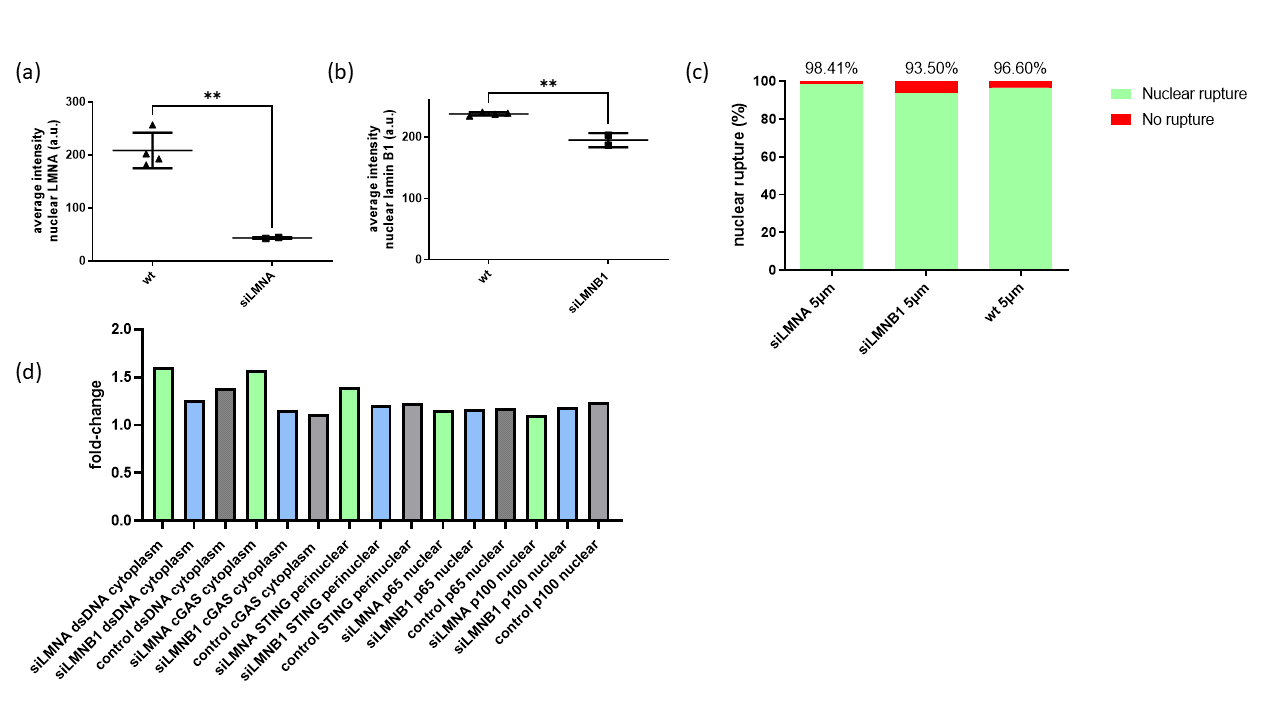


**Fig. S10. Effect of lamins in nuclear rupture and in the activation of cGAS/STING in the cell line MDA-MB-231 induced by capillary constriction forces.** (a) Average nuclear intensity of lamin A/C in wild type and siLMNA cells. (b) Average nuclear intensity of lamin B1 in wild type and siLMNB1 cells. Each dot represents a technical replicate, in which at least 400 cells per condition were quantified. (c) Fraction of NLS-GFP MDA-MB-231 siLMNA, NLS-GFP MDA-MB-231 siLMNB1 and wt NLS-GFP MDA-MB-231 cells experiencing nuclear envelope rupture while transiting 5µm-constrictions under physiological pressure, (d) fold-increase of dsDNA cytoplasm, cGAS cytoplasm, STING perinuclear, p65 and p100 nuclear of siLMNA, siLMNB1 and wt cells that have transited 5μm constrictions compared to untransited cells. Each group was composed of a minimum of n=200 cells. Comparisons among groups were performed by unpaired t test, two-tailed (t=46.48, DF=6, ∗p < 0.05,∗∗p < 0.01,∗∗∗p < 0.001,∗∗∗∗p < 0.0001).


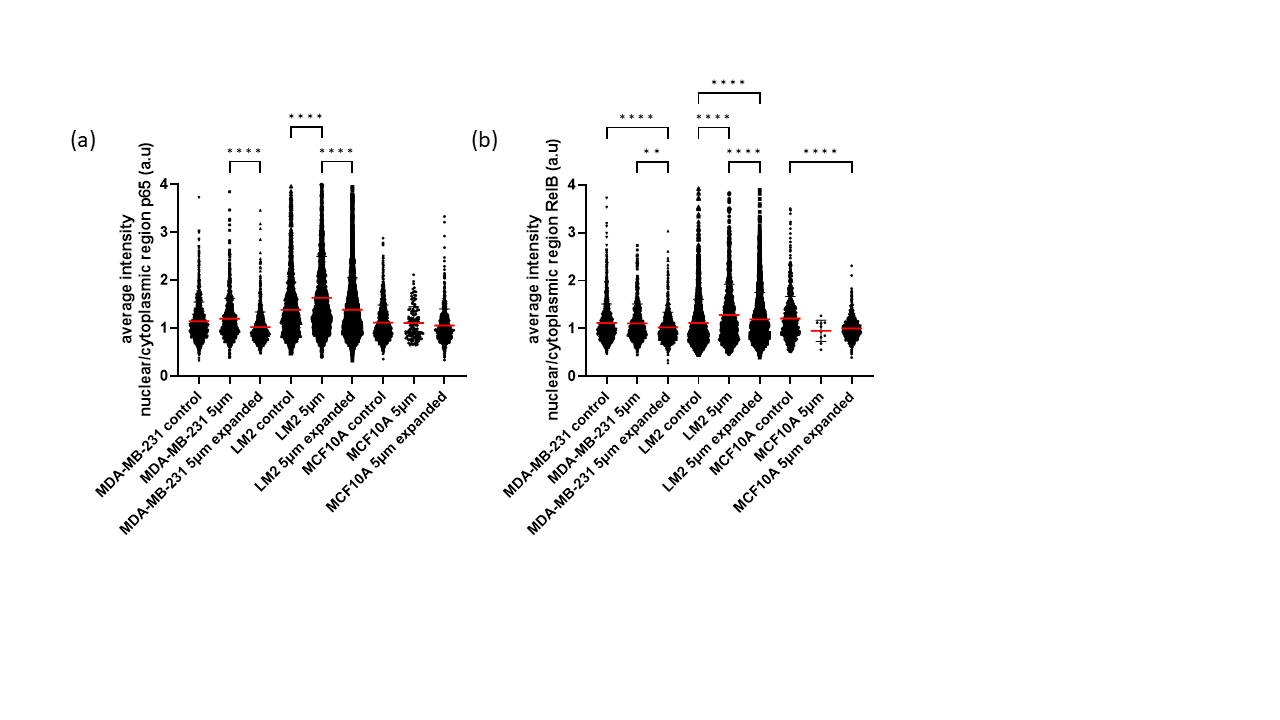


**Fig. S11. Nuclear translocation ratios of p65 and RelB.** Nuclear/cytoplasmic region ratio of the average intensity of p65 (a) and RelB (b) in MDA-MB-231, LM2 and MCF-10A cells. The group “5µm” refers to cells that have transited through 5x5µm^2^ constrictions, the group “5µm expanded” refers to cells that have transited 5x5µm^2^ constrictions and have been expanded for 2-5 passages to provide them sufficient time to recover and the group “control” refers to cells that have not transited the constrictionEach dot represents a cell. For each group, means are highlighted in red. Within each cell line, comparisons among groups were performed by one-way ANOVA (Tukey multiple comparison test;∗p < 0.05,∗∗p < 0.01,∗∗∗p < 0.001,∗∗∗∗p < 0.0001).


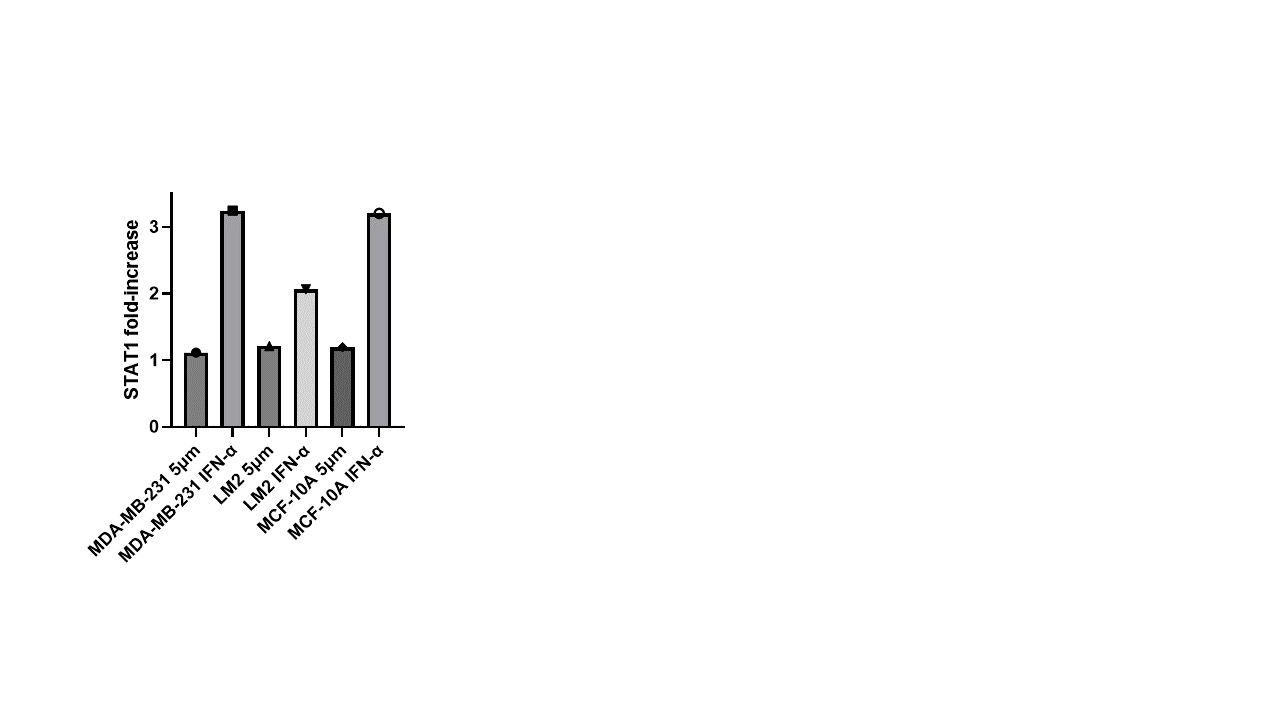


**Fig. S12. Fold increase of STAT1 activation.** Fold increase of STAT1 nuclear average intensity in MDA-MB-231, LM2 and MCF-10A cells after treating the cells with IFN-α and after cells transited 5μm constrictions. Fold increase was calculated with respect to controls for each condition, i.e., cells that did not transit the constriction and cells that were not treated with IFN-α, respectively.


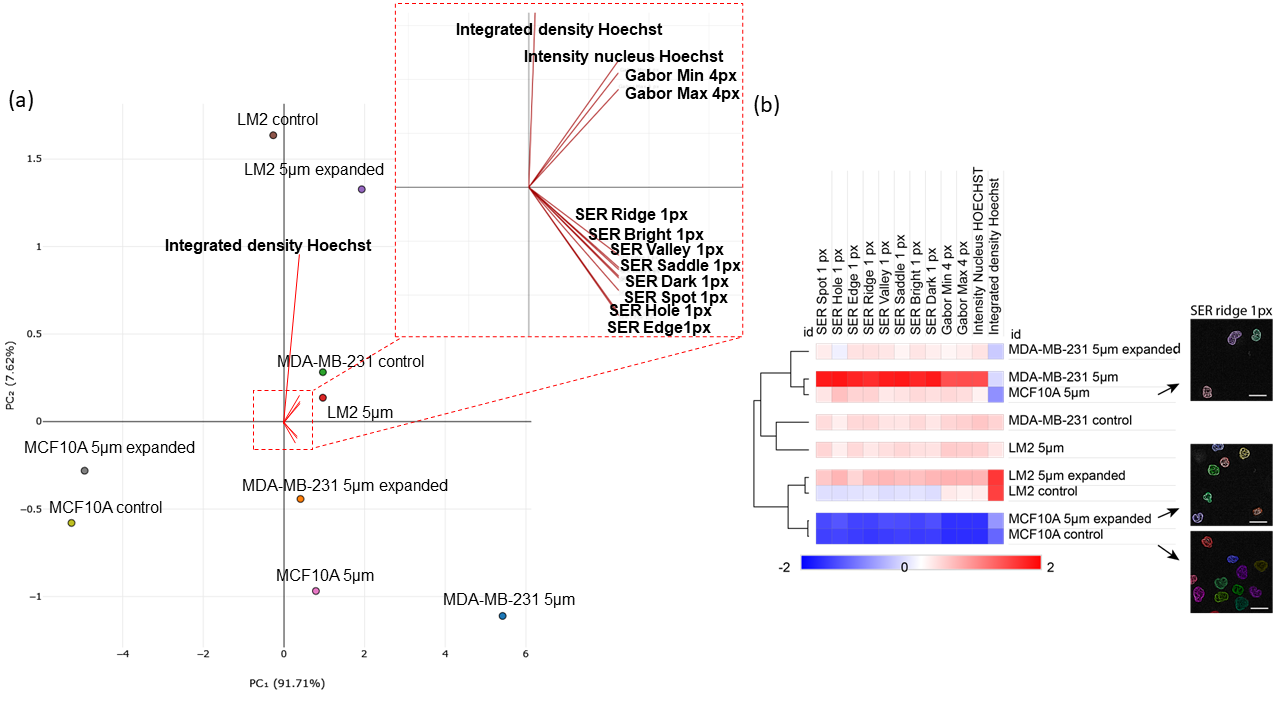


**Fig. S13. Evaluation of chromatin condensation state by Hoechst intensity and texture quantification**. Representation of the PCA space (PCA1 and PC2) of SER (Spot, Hole, Edge, Ridge, Valley, Saddle, Bright, Dark), Gabor (min, max) and intensity (integrated density, average intensity) features of nuclear Hoechst signal in MDA-MB-231, LM2 and MCF-10A cells. Loading vectors are presented in red. (b) Hierarchical clustering of the MDA-MB-231, LM2 and MCF-10A cells based on these features, including a representative image of SER ridge texture of MCF-10A cells of each group. Scale bar: 20µm. The group “5µm” refers to cells that have transited through 5x5µm^2^ constrictions, the group “5µm expanded” refers to cells that have transited 5x5µm^2^ constrictions and have been expanded for 2-5 passages to provide them sufficient time to recover and the group “control” refers to cells that have not transited the constriction.

**
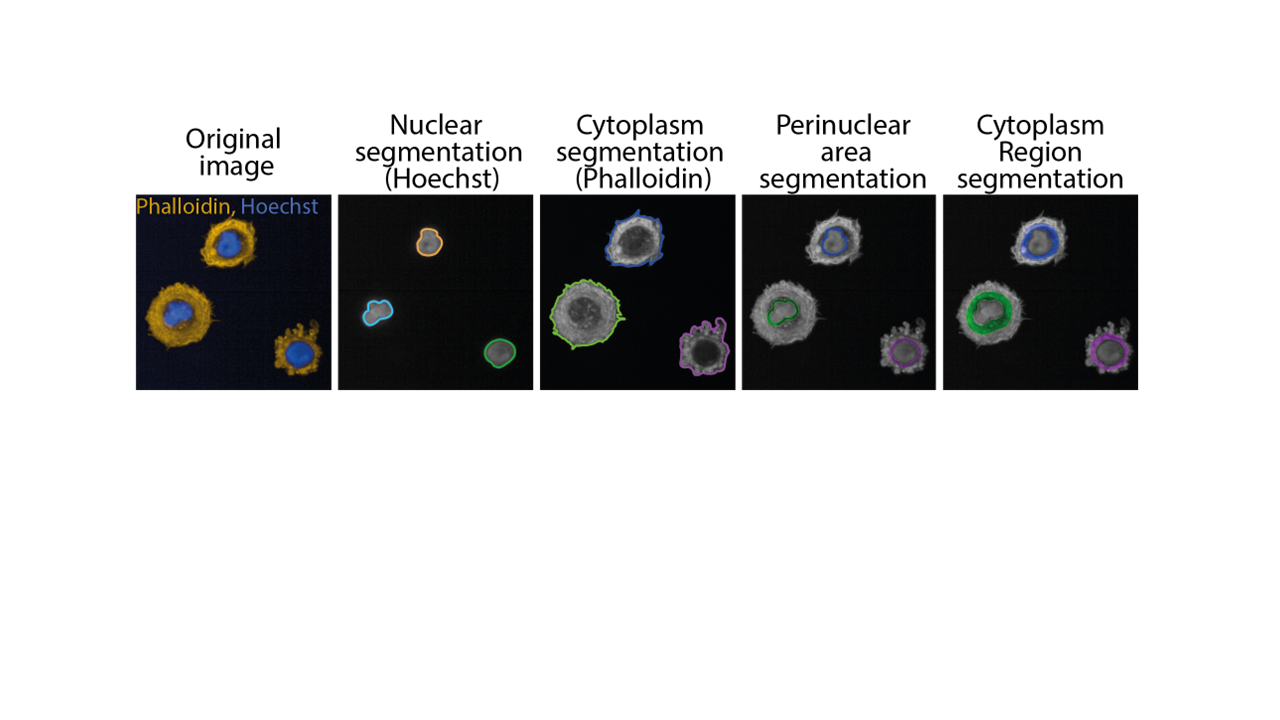
**

**Fig. S14. Example of a cell segmentation using Harmony High-Content Analysis software.** Maximum intensity projection of a confocal image (original image). Hoechst was used for nuclei segmentation and Phalloidin for cytoplasm segmentation, followed by the segmentation of perinuclear area and cytoplasm region.

Supplementary Tables

**Supplementary Table 1. RNA sequencing experiment details**

| **Experiment** | **Number of chips** | **Cell concentration** | **Total RNA amount (ug)** |
| --- | --- | --- | --- |
| MDA-MB231 Replicate 1 | 19 | Control: 3.03x10^6^ cells/ml in 1ml. Constriction: 3.54x10^5^ cells/ml in 200µl | Control: 11.20688  Constriction: 0.05103 |
| MDA-MB231 Replicate 2 | 16 | Control 1.09x10^6^ cells/ml in 1ml. Constriction 5.7x10^5^ in 200µl | Control: 5.81922  Constriction: 0.13779 |
| LM2 Replicate 1 | 18 | Control 1.3 x10^6^ cells/ml in 1ml. Constriction 8.95 x10^5^ in 200µl | Control: 6.00396  Constriction: 0.09643 |
| LM2 Replicate 2 | 18 | Control 6.61x10^5^ cells/ml in 1ml. Constriction 8.23x10^5^ in 200µl | Control: 3.57298  Constriction: 0.23839 |
| MCF-10A Replicate 1 | 18 | Control 7.08x10^6^ cells/ml in 1ml (full T25 flask).  Constriction 5.27x10^5^ cells/ml in 200ul | Control: 48.15319  Constriction: 32.63773 |
| MCF-10A Replicate 2 | 20 | Control 5.89x10^5^ cells/ml. Constriction 2.73x10^5^ cells/ml in 200ul | Control: 1.02540  Constriction: 4.46207 |

**Supplementary Table 2. Primary antibodies used for immunofluorescence.**

| **Target** | **Antibody and species** | **Company and cat number** | **WD Dilution** |
| --- | --- | --- | --- |
| STING | Rabbit | Abcam ab181125 | 1:100 |
| cGAS | Rabbit | Sigma HPA031700 | 1:100 |
| dsDNA | Mouse | Abcam ab27156 | 1:500 |
| NF-kB p65 | Rabbit | Abcam ab16502 | 1:200 |
| RelB | Rabbit | Abcam ab33907 | 1:100 |
| Anti-Lamin A | Mouse | Abcam ab8980 | 1:400 |
| Ani-lamin B | Rabbit | Ab16048 | 1:500 |
| Tubulin-alpha | Rat | BioRad YOL1/34 | 1:500 |
| Phospho IRF-3 | Rabbit | CellSignal mAb #29047 | 1:100 |
| Phospho-STAT 1 | Rabbit | CellSignal mAb #9167 | 1:500 |
| STAT3 | Mouse | CellSignalling mAb #9139 | 1:500 |
| Ki67 | Rabbit | Abcam ab16667 | 1:250 |
| Phalloidin iFluor 555 | N/A | ab176756 | 1:500 |

**Supplementary Table 3.** **Downregulated genes differentially expressed in MDA-MB-231, LM2 and MCF-10A after transiting constrictions.**

| **Downregulated in:** | **Gene name** | **Gene biotype** | **Gene description** |
| --- | --- | --- | --- |
| MDA-MB231, MCF-10 and LM2 | *RPL39P3* | processed pseudogene | 60S ribosomal protein L39 |
| MDA-MB231 and MCF-10 | *AC004453.1* | processed pseudogene | 60S ribosomal protein L32 |
|  | *AC004771.5* | antisense | Novel transcript, antisense to CAMTA2 |
|  | *AC010487.2* | lincRNA | novel transcript |
|  | *AC044849.1* | antisense | Store-operated calcium entry-associated regulatory factor |
|  | *AC135178.3* | antisense | novel transcript, antisense to KRBA2 and RPL26 |
|  | *AC145098.1* | antisense | novel transcript |
|  | *AL662791.1* | antisense | HLA complex group 16 |
|  | *EGR2* | protein coding | Zinc finger protein Krox-20 |
|  | *LAMB4* | protein coding | Laminin beta-1-related protein |
|  | *MATN2* | protein coding | matrilin 2 |
|  | *MT-TT* | Mt tRNA | mitochondrially encoded tRNA threonine [Source:HGNC Symbol;Acc:HGNC:7499] |
|  | *RF00019* | misc RNA | non-coding RNA |
|  | *TPT1-AS1* | processed transcript | Reticulocalbin-1 |
| MCF-10A and LM2 | *AC025857.2* | sense intronic | novel transcript, sense intronic to CTSB |
|  | *C5AR2* | protein coding | G-protein coupled receptor 77 |
|  | *CLTRN* | protein coding | Collectrin |
| MDA-MB-231 and LM2 | *ATF3* | protein coding | Cyclic AMP-dependent transcription factor ATF-3 |
|  | *EEF1A1P6* | processed pseudogene | Eukaryotic elongation factor 1 A-1 |
|  | *RPL26* | processed pseudogene | Silica-induced gene 20 protein |

**Supplementary Table 4.** **Upregulated genes differentially expressed in MDA-MB-231, LM2 and MCF-10A after transiting constrictions.**

| **Upregulated in:** | **Gene name** | **Gene biotype** | **Gene description** |
| --- | --- | --- | --- |
| MDA-MB231, MCF-10 and LM2 | *AL355032.1* | processed pseudogene | Silica-induced gene 20 protein |
|  | *MT-ATP8* | protein coding | F-ATPase subunit 8 |
|  | *RN7SL2* | misc RNA | RNA, 7SL, cytoplasmic 2 |
| MDA-MB231 and MCF-10 | *AC004898.1* | processed pseudogene | p97 cofactor p37 |
|  | *Z95331.1* | sense overlapping | Uncharacterized protein C9orf85 |
|  | *ZNF460* | protein coding | Zinc finger protein HZF8 |
| MDA-MB-231 and LM2 | *AP000866.6* | sense intronic | Endothelial cell-selective adhesion molecule |
|  | *HIST1H1E* | protein coding | Histone H1b |
|  | *HIST1H4A* | protein coding | Histone H4 |
|  | *TMEM238* | protein coding | Putative transmembrane protein LOC100289255 |
|  | *TYRO3P* | processed pseudogene | TYRO3P protein tyrosine kinase pseudogene |
